# Acylcarnitines promote gallbladder cancer metastasis through lncBCL2L11-THOC5-JNK axis

**DOI:** 10.1186/s12967-024-05091-0

**Published:** 2024-03-22

**Authors:** Yang Yang, Huaifeng Li, Ke Liu, Lu Zou, Shanshan Xiang, Yajun Geng, Xuechuan Li, Shimei Qiu, Jiahua Yang, Xuya Cui, Lin li, Yang Li, Weijian Li, Siyuan Yan, Liguo Liu, Xiangsong Wu, Fatao Liu, Wenguang Wu, Shili Chen, Yingbin Liu

**Affiliations:** 1 Department of Biliary-Pancreatic Surgery, Renji Hospital Affliated to Shanghai Jiao Tong University School of Medicine, Shanghai, 200127 China; 2 Department of General Surgery, Xinhua Hospital Affiliated to Shanghai Jiao Tong University School of Medicine, Shanghai, 200092 China; 3 University of Shanghai for Science and Technology, Shanghai, 200093 China

**Keywords:** Acylcarnitine, Gallbladder cancer, lncBCL2L11, Lipid

## Abstract

**Background:**

The progression of gallbladder cancer (GBC) is accompanied by abnormal fatty acid β-oxidation (FAO) metabolism. Different types of lipids perform various biological functions. This study aimed to determine the role of acyl carnitines in the molecular mechanisms of GBC progression.

**Methods:**

Distribution of lipids in GBC was described by LC–MS-based lipidomics. Cellular localization, expression level and full-length of lncBCL2L11 were detected using fluorescence in situ hybridization (FISH) assays, subcellular fractionation assay and 5′ and 3′ rapid amplification of the cDNA ends (RACE), respectively. In vitro and in vivo experiments were used to verify the biological function of lncBCL2L11 in GBC cells. Methylated RNA Immunoprecipitation (MeRIP) was performed to detect the methylation levels of lncBCL2L11. RNA pull-down assay and RNA immunoprecipitation (RIP) assay were used to identify lncBCL2L11 interacting proteins. Co-Immunoprecipitation (Co-IP) and Western blot assay were performed to validate the regulatory mechanism of lncBCL2L11 and THO complex.

**Results:**

Acylcarnitines were significantly up-regulated in GBC tissues. High serum triglycerides correlated to decreased survival in GBC patients and promoted tumor migration. LncBCL2L11 was identified in the joint analysis of highly metastatic cells and RNA sequencing data. LncBCl2L11 prevented the binding of THOC6 and THOC5 and causes the degradation of THOC5, thus promoting the accumulation of acylcarnitines in GBC cells, leading to the malignant progression of cancer cells. In addition, highly expressed acylcarnitines stabilized the expression of lncBCL2L11 through N^6^-methyladenosine methylation (m6A), forming a positive feedback regulation in tumor dissemination.

**Conclusions:**

LncBCL2L11 is involved in gallbladder cancer metastasis through FAO metabolism. High lipid intake is associated with poor prognosis of GBC. Therefore, targeting lncBCL2L11 and its pathway-related proteins or reducing lipid intake may be significant for the treatment of GBC patients.

**Supplementary Information:**

The online version contains supplementary material available at 10.1186/s12967-024-05091-0.

## Background

Gallbladder cancer is a common biliary system malignancy with a high mortality rate. Patients with advanced GBC have extremely poor prognosis with a median survival period of 5.2–24.4 months [1–5]. Despite novel clinical efforts, including targeted therapy and immunotherapy for GBC, satisfactory results are yet to be achieved. Radical resection remains the only effective treatment currently available owing to GBC chemoresistance [6–8]. However, radical resection is directly restricted (25%) in patients presenting with symptomatic and more advanced disease [9–11], including the presence of metastasis, contiguous organ involvement, and vascular invasion. Therefore, identifying the molecular mechanisms of GBC metastasis is essential for GBC treatment.

Cancer cells exhibit unique metabolic adaptations during their malignant progression [12, 13]. In addition to the abnormal glucose and nucleic acid metabolism, some tumors display dysregulated fatty acid β-oxidation (FAO) metabolism, and the products act as a fuel or cell membrane component to support tumor survival and a signaling molecule that participates in the regulation of cancer signaling pathways. Growing evidence demonstrates the importance of FAO in cancer cell survival, proliferation, stemness, drug resistance, and metastasis [14–17].

Long non-coding RNAs (lncRNAs) are a class of RNA transcripts greater than 200 nucleotides in length with no protein-coding capacity. Studies have shown that lncRNAs can affect FAO activity in pathological and physiological states. For example, the lncRNA HCP5 has been identified as an important FAO regulator that promotes stemness and chemoresistance in gastric cancer cells [18], and mesenchymal stem cell culture-induced AGAP2-AS1 causes stemness and trastuzumab resistance via promoting CPT1 expression and inducing FAO in breast cancer [19]. Previous studies have reported abnormal lipid metabolism in GBC [20–22], indicating that lipid metabolism reprogramming is critical for the survival and proliferation of tumor cells. However, the potential functions of lipids and their regulatory mechanisms in GBC remain unknown.

This study uncovered a role of acylcarnitines in hepatic metastasis of GBC and a novel mechanism of lncBCL2L11 in regulating FAO metabolism. Our findings provide a mechanistic insight into GBC metastasis. Low-fat diets or therapies targeting this regulatory axis may have important implications for GBC prevention and even treatment.

## Methods

### Cell lines

Human GBC cell lines NOZ were obtained from the Health Science Research Resources Bank (Osaka, Japan). GBC-SD cells were supplied by Shanghai Institute for Biological Science, Chinese Academy of Science (Shanghai, China). ZJU-0430 cells were purchased from MingZhou Biological Company (Zhejiang, China). All cells were cultured with high-glucose DMEM medium (Gibco) supplemented with 10% fetal bovine serum (Gibco)in a 5% CO_2_ incubator at 37 °C (Thermo Fisher Scientific, Massachusetts, USA). All the cell lines were identified by Short Tandem Repeat (STR) sequencing.

### Transwell assay

Transwell assay was performed through an 8 μm sized filter (BD Biosciences) to detect the in vitro migration ability of GBC cells. In short, cells (2 × 10^4^) in 200 μL serum-free medium were added to the upper chamber and the low chamber was filled with 600 μL medium containing 10% FBS. After 16 h of culture, cells migrated to the lower layer were fixed with 4% paraformaldehyde and stained with crystal violet. Bottom cells were finally counted under a microscope, reflecting migration ability.

### Animals

BALB/c nude mice (Male, 4–5 weeks old) were purchased from the Shanghai Laboratory Animal Center of the Chinese Academy of Sciences (Shanghai, China) and randomly divided into different groups (4–5 mice per group to satisfy different request). The subcutaneous xenotransplantation model was established by subcutaneous injection of 10^6^ cells suspension into the armpit of nude mice. High fat diet (D12492; Research Diets, Inc.) feeding began in 6-week-old mice. About 4 weeks later, the mice were sacrificed and the tumors were harvested and weighed. The intrasplenic injection model was referred to previous operations. 10^6^ cells after different treatment were injected into the spleen of mice at homogenization rate. Then, the spleen was compressed for 2 min followed by a splenectomy. After 4 weeks, the liver metastases were collected. All the tumors were stored and fixed in 4% paraformaldehyde for further assays.

### Quantitative real-time PCR (qRT-PCR)

Total RNA from cell or tissues was extracted using TRIzol reagent (Invitrogen). The gDNA Eraser and PrimeScript™ RT reagent Kit (Takara) was used for synthesis of cDNA and SYBR^®^ Premix Ex Taq™ II (Takara) was used for qRT-PCR. GAPDH was used as an endogenous control. Primers were listed in additional file (Additional file 1: Table S4).

### Patient specimens

The study protocol was approved by the ethics committee of Xinhua Hospital, Shanghai Jiao Tong University School of Medicine. Human GBC tissues and adjacent benign gallbladder tissues (at least 2 cm away from the tumor) were obtained from patients who received cholecystectomy without accepting any preoperative treatment.

### RNA pull-down and mass spectrometry

RNA pull-down assays were performed according to the previous instructions. Briefly, biotin-labeled lncRNA was first transcribed in vitro and incubated with streptavidin dynabeads at 4 °C for 1 h. Then, the RNA-beads complex was incubated with cell lysates for 3.5 h at 4 °C. Interacting proteins were further eluted by SDS loading buffer and separated by sodium dodecyl sulphate–polyacrylamide gel. The specific binding protein band was identified by silver stain and submit to mass spectrometry analysis.

### RNA immunoprecipitation (RIP)

RNA immunoprecipitation assays were performed using the Megna RIP RNA-binding Protein Immunoprecipitation Kit (Millipore, Massachusetts, USA). The Interacting RNAs were detected by both quantitative real-time PCR and reverse transcription PCR.

### Isolation of cytoplasmic and nuclear RNA

RNA isolation from cytosolic and nuclear fractions was using the PARIS™ Kit (Thermo Fisher Scientific, Massachusetts, USA) according to the manufacturer’s instructions.

### Western blot assay

Cells or tissues were lysed on ice using RIPA buffer (Beyotime, Shanghai, China) and quantified by Bicinchoninic Acid Kit. Proteins were separated by 10% or 12% polyacrylamide gel and blotted to a PVDF membrane (Millipore, Darmstadt, Germany). The primary antibody was incubated following the instructions, and the secondary antibody was incubated for 1 h at room temperature. Antibodies were listed in the Additional file 1: Table S2.

### 5′ and 3′ Rapid amplification of cDNA ends (5′ 3′ RACE)

Total RNA from NOZ cells was isolated using TRIzol Plus RNA Purification Kit (Thermo Fisher Scientific, Massachusetts, USA), following the manufacturer’s protocols. RACE assays were performed using GeneRacer™ Kit (Thermo Fisher Scientific, Massachusetts, USA) according to the manufacturer’s instructions. Gene primers were listed in Additional file 1: Table S4.

### Fluorescence in situ hybridization (FISH)

Fluorescence in situ hybridization assay was performed according to the manufacturer’s instructions (GenePharma, shanghai, China). The probe used for lncBCL2L11 is listed in Additional file 1: Table S5.

### Immunohistochemistry (IHC)

Immunohistochemical staining was performed using a standard immunoperoxidase staining procedure.

### Co-inmunoprecipitation (Co-IP)

Protein A/G magnetic beads (Bio-Rad, California, USA) were first precleared and incubated with corresponding antibodies. Then, cell lysates were extracted and incubated with antibody-beads complex at 4 °C overnight after transfected with different plasmids or siRNAs. The beads were finally washed with lysis buffer three times and the proteins were detected by western blot assay.

### Bioinformatics analysis

Human microarray containing 9 paired gallbladder cancer and adjacent tissues was downloaded from the NCBIs Gene Expression Omnibus (GEO, GSE76633). Lim2-NOZ microarray was obtained from GSE106671. Differentially expressed genes were identified by limma package (version 3.48.3). Gene Ontology and Kyoto Encyclopedia of Genes and Genomes (KEGG) enrichment analyses were performed by the clusterProfiler package. The gene sets collection (h.all.v7.4.entrez.gmt) from the Molecular Signatures Database (MsigDB) was used for the enrichment analysis.

### Lipids extraction

Lipids from tissues or cells were extracted using a combination of MTBE based liquid extraction protocol. As described previously, 100 μL sample lysis was extracted twice with 300 μL methanol and 1 mL MTBE. After shaking for 1 h, 250 μL of water were added to the mixture. The extract solvent was centrifuged at 4 ℃ for 10 min at 14,000*g*, to induce the phase separation, and the supernatant mainly containing lipophilic metabolites. After the samples were dried by the Laboconco Vacuum Dry Evaporator. 120 μL of acetonitrile/isopropanol/water (65/30/5) solvent was added to reconstitute the dried extract for subsequent lipidomics analysis.

### LC–MS-based lipidomics

Lipidomics analysis was performed on ultra-high performance liquid chromatography (UHPLC, Dionex) coupled with high resolution quadrupole-orbitrap mass spectrometry (UHPLC-Q-Exactive plus, ThermoFisher). The lipid extract was separated on a BEH C8 column (1.7 μm, 2.1 × 100 mm). The column temperature was maintained at 55 0C. Mobile phase A was 60% acetonitrile/40% water, mobile phase B was 90% isopropanol/10% acetonitrile, both containing 10 mmol/L ammonium acetate, and the flow rate was 0.26 mL/min. The gradient starts from 32% B and maintains for 1.5 min, linearly increases to 85% B at 15.5 min, to 97% B at 15.6 min and maintains for 1.4 min, decrease to 32% B at 18.1 min and maintains for another 2.9 min to equilibrate the column. The total gradient time was 21 min.

The mass spectrometry was operated in positive and negative ion modes, respectively. For the positive ion mode, the spray voltage was 3500 V, the capillary temperature was 300 °C, the aux gas heater temperature was 350 °C, the sheath gas flow rate was 45, the aux gas flow rate was 10, the S-lens RF level was 50. The mass resolution of full-scan ms was 70,000, the m/z scan range was 150–1500. Precursor ion with the top 10 intensities were automatically selected for data-dependant MS/MS with the mass resolution of 17,500. For the negative ion mode, the spray voltage was 3000 V, the capillary temperature was 320 °C, the aux gas heater temperature was 350 °C, the sheath gas flow rate was 35, the aux gas flow rate was 8, the S-lens RF level was 50, with the other parameters same as in the positive ion mode.

### Data pre-processing

A peak table containing the retention time, m/z, peak area, and lipid identification results was obtained by peak matching and structural identification using the MS-DIAL software [23]. Briefly, the raw MS data were converted to the common file format of Reifycs Inc. (.abf) using the Reifycs ABF converter. After that, MS-DIAL software was used for feature detection, spectra deconvolution, lipid identification, and peak alignment among samples. MS/MS spectra-based lipid identification was performed in MS-DIAL software by searching the acquired MS/MS spectra against the internal in silico MS/MS spectra database. It includes MS1 and MS/MS information of common lipid species. Tolerances for MS1 and MS/MS searches were set to 0.01 and 0.05 Da, respectively.

### Statistical analysis

Statistical analysis was carried out by SPSS 19.0 software using student’s t tests or one-way analysis of variance when necessary, and the data were shown as the mean ± SD. Pearson *χ*^2^ test was used to analyze the association between lncBCL2L11 expression and clinicopathological variables. Kaplan‐Meier method and log‐rank tests were used for the survival analysis. All *p* values were two-sided unless otherwise stated and *p* value < 0.05 was considered to be statistically significant.

## Results

### Accumulation of acylcarnitines promotes GBC migration

**Fig. 1:**
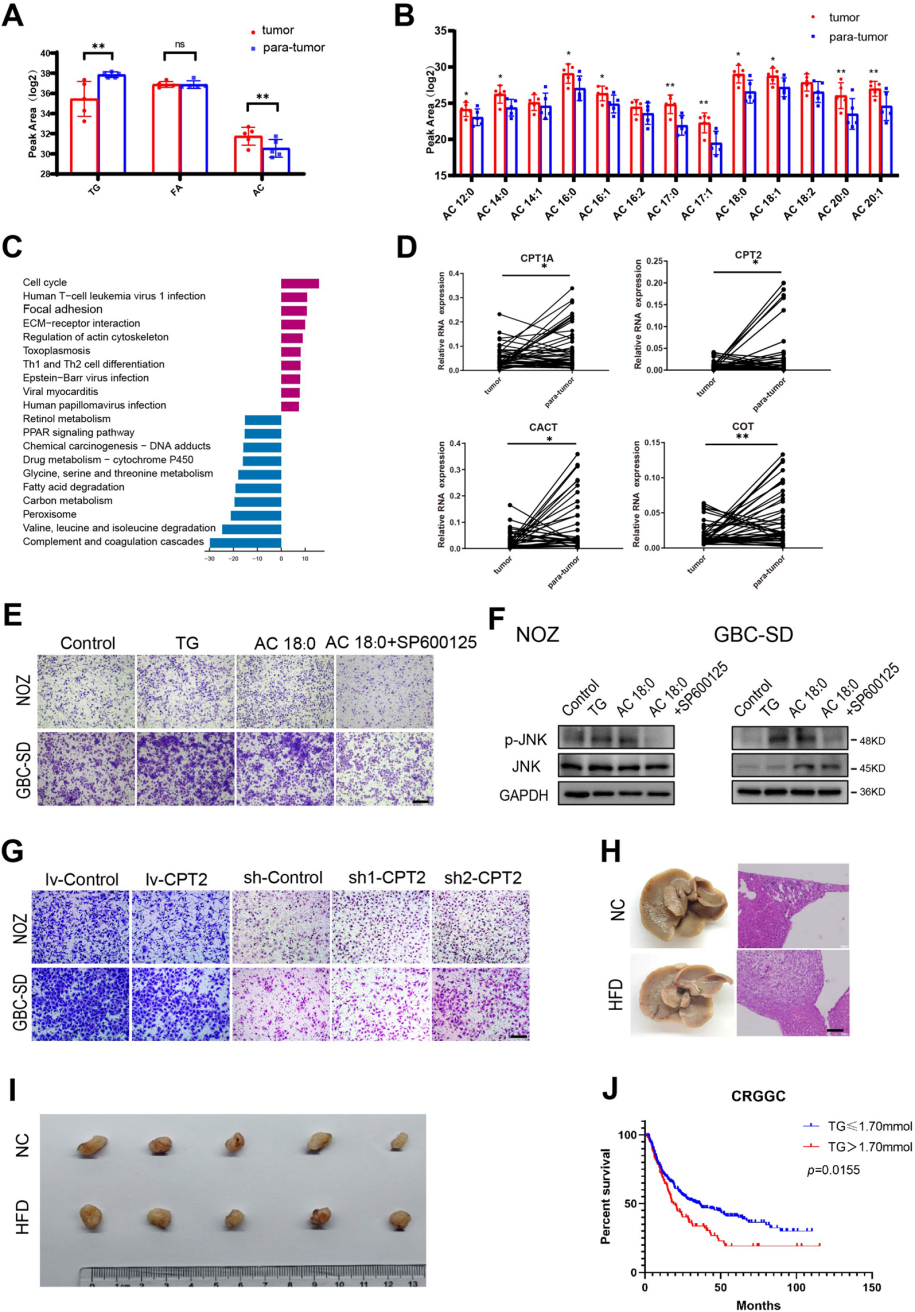
Accumulation of acylcarnitines promotes GBC migration. **A** Lipids were detected by LC–MS-based lipidomics in gallbladder carcinoma and para-tumor tissues. **B** Different types of acylcarnitines (ACs) in GBC tissues. **C** KEGG enrichment analysis of differential genes between cancer and adjacent tissues (GSE76633). **D** Expression of FAO related genes in paired GBC and adjacent tissues. **E** Transwell assays were performed in NOZ and GBC-SD cells cocultured with triglycerides, acylcarnitines and JNK inhibitor SP600125. Scale bars, 100 μm. **F** Protein levels of p-JNK and JNK in NOZ and GBC-SD cells after different treatment. **G** Transwell assays were used in NOZ and GBC-SD cell after CPT2 overexpression and knockdown. Scale bars, 100 μm. **H** Representative images of liver metastasis after 4-week high fat intake(left). Liver tissues section were stained by hematoxylin and eosin(right). Scale bars, 100 μm. **I** Images of tumor formation in nude mice after 4-week high fat intake. **J** Kaplan–Meier survival curves of 322 GBC patients with different levels of serum triglycerides

FAO is a major source of bioenergy and recently is considered a prominent hallmark of cancer. To assess the effect of lipid metabolism on GBC progression, various lipid s in GBC tissues were analyzed using LC–MS-based lipidomics (Additional file 1: Table S1). The results showed a significant decrease in total triglycerides and an increase in acylcarnitines in tumor tissues compared to para-tumor tissues (Fig. 1A and Additional file 2: Fig. S1A, B). Subcomponent analysis showed an overall increase in acylcarnitines rather than triglycerides and fatty acids (Fig. 1B and Additional file 2: Fig. S1C, D). Changes in lipid composition imply alterations in metabolic pathway. To elucidate lipid-related genes in GBC tumorigenesis, microarray analysis was performed to profile the gene expression of nine paired tumor tissues and adjacent tissues (GES76633) [24]. Gene ontology enrichment and KEGG analyses showed a significant decrease in FAO in GBC tissues (Fig. 1C and Additional file 2: Fig. S2A). Real-time PCR (RT-PCR) was performed in 40 new paired cases of GBC to validate the bioinformatics analysis results. Consistent with the high-throughput data, key enzymes in FAO, such as carnitine palmitoyltransferase I (CPT1A), carnitine palmitoyltransferase II (CPT2), carnitine-acylcarnitine translocase (CACT), and mitogen-activated protein kinase kinase kinase 8 (MAP3K8, COT), were significantly decreased (Fig. 1D and Additional file 2: Fig. S2B). Low expression of FAO related genes was also found in another dataset (GES139683) (Additional file 2: Fig. S2B). These data suggest that FAO metabolism in GBC tissues was significantly downregulated.

Given the reduction in lipid metabolism in GBC and the increase in acylcarnitines, we speculate acylcarnitines (whether caused by endogenous metabolic production or exogenous uptake) play an important role in oncogenesis. So, the biological function of acylcarnitines in GBC was clarified. Tumor cells co-cultured with stearyl carnitine (acylcarnitine 18:0) and triglycerides showed enhanced cell migration (Fig. 1E) and increased phosphorylation of c-Jun NH2-terminal kinases (p-JNK), which is a powerful stress-induced protein regulating lipid metabolism and an important promoter in GBC (Fig. 1F) [25–28]. Cell migration ability was significantly reduced after JNK inhibition by SP60015, which indicated that lipids promoted GBC metastasis through the JNK pathway. To further confirm these findings, endogenous acylcarnitines was altered by regulating the expression of CPT2. We observed that CPT2 overexpression decreased the content of acylcarnitines and inhibited cell migration, and CPT2 knockdown promoted metastasis of GBC (Fig. 1G). In vivo, the increased consumption of high-fat diet (HFD, 60% kcal fat) showed no influence on tumor proliferation but significantly promoted liver metastasis (Fig. 1H, I and Additional file 2: Fig. S2D). These data suggest that a decrease in FAO or an increase in both endogenous and exogenous acylcarnitines promotes GBC cell migration.

Consistent with in vivo experiments, we analyzed 332 patients with complete biochemical indices and follow-up information from the Chinese GBC database of the Chinese Research Group of Gallbladder Cancer [25] and 88 advanced GBC patients from RJ corhort. High serum triglycerides (> 1.70 mmol/L) were found to be closely associated with tumor invasion and metastasis (Tables 1 and 2). GBC patients with high triglyceride levels had significantly shorter overall survival than those with low triglyceride levels (≤ 1.70 mmol/L) (Fig. 1J). Collectively, our data demonstrate that acylcarnitines and TG act as a tumor-promoting factor in GBC cells.

**Table 1:** Association of triglyceride levels and clinicopathological characteristics of 332 GBC patients from CRGGC

Characteristic	No. of cases	TG(mmol/L)		*χ*^2^	p value
		≤ 1.70	> 1.70		
Age					
≤ 60	120	84 (70.0)	36 (30.0)		
> 60	212	146 (68.9)	66 (31.1)	0.046	0.830
Gender					
Male	128	90 (70.3)	38 (29.7)		
Female	204	140 (68.6)	64 (31.4)	0.105	0.746
Tumor invasion					
T1	31	27 (87.1)	4 (12.9)		
T2	81	50(61.7)	31 (38.3)		
T3	200	143 (71.5)	57 (28.5)		
T4	20	10 (50.0)	10 (50.0)	10.75	0.013*
Lymph node metastasis					
N0	178	127 (71.3)	51 (28.7)		
N1	119	78 (65.5)	41 (34.5)		
N2	35	25 (71.4)	10 (28.6)	1.213	0.545
Metastasis					
M0	304	215 (70.7)	89(29.3)		
M1	28	15 (53.5)	13 (46.4)	3.544	0.06

Tumor staging according to AJCC 8th edition，* indicates statistical significance

**Table 2:** Association of triglyceride levels and clinicopathological characteristics of 88 advanced GBC patients from RJ corhort

Characteristic	No. of cases	TG(mmol/L)		*Χ*^2^	p value
		≤ 1.70	> 1.70		
Age					
≤ 60	62	34 (54.8)	28 (45.2)		
> 60	26	15 (57.7)	11 (42.3)	0.060	0.807
Gender					
Male	35	20 (57.1)	15 (42.9)		
Female	53	29 (54.7)	24 (45.3)	0.050	0.823
Liver metastasis					
No	65	42 (64.6)	23(35.4)		
Yes	23	7 (30.4)	16 (69.6)	8.043	0.005*

Tumor staging according to AJCC 8th edition，* indicates statistical significance

### lncBCL2L11 associated with tumor metastasis is screened in both cell lines and tumor tissues

**Fig. 2:**
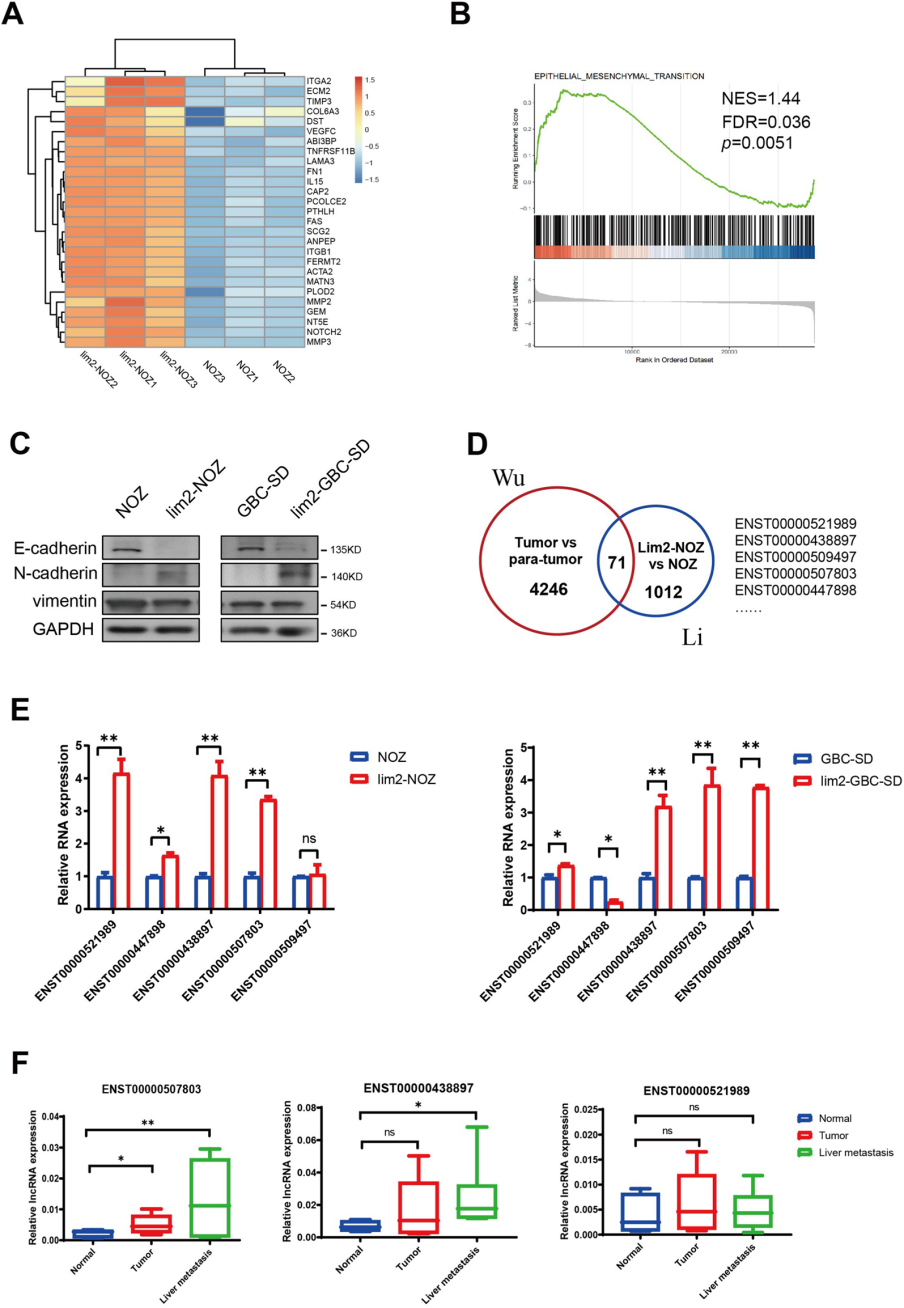
Screening lncRNAs associated with tumor metastasis in relatively high metastatic cell lines. **A** GSEA analysis was used to identify differential genes profiles between lim2-NOZ and NOZ cells. **B** Up-regulated genes in EMT pathway were found in lim2-NOZ compared to NOZ cells. **C** Protein levels of N-cadherin, E-cadherin, and Vimentin in lim2 and parent cells. **D** Co-upregulated lncRNAs in two high-throughput data were showed by Venn diagram. **E** Candidate lncRNAs were verified in lim2-NOZ and lim2-GBC-SD cells. **F** Three candidate lncRNAs were detected in 6 paired GBC tissues, including normal tissues, tumor tissues and liver metastases (Paired t test)

In a previous study, relatively high metastatic cell lines, lim2-NOZ and lim2-GBC-SD, were established via reduplicate splenic administration with GBC cells. The enhanced migration ability of the cells has been demonstrated using in vivo and in vitro functional experiments [26]. To further confirm the migration mechanism in these two cell lines, we re-analyzed the microarray data of lim2-NOZ and parental NOZ cells (GSE106671). Gene set enrichment analysis revealed that a series of upregulated genes were enriched in the epithelial-mesenchymal transition pathway in lim2-NOZ cells (Fig. 2A and B). Western blotting confirmed the downregulation of E-cadherin and upregulation of N-cadherin in both cell lines (Fig. 2C).

Acylcarnitines cause liver metastasis of GBC; thus, the specific molecules associated with GBC migration and FAO metabolism were further assessed. Two high-throughput datasets (GSE106671 and GES76633) were overlapped to screen lncRNAs associated with liver metastasis and FAO metabolism of GBC. Strict screening criteria (lncRNA length > 400 bp, foldchange > 2, p-value < 0.05, and efficient amplification in PCR assay) were set to enable the convenient and effective filtering of potential lncRNAs. Candidates significantly overexpressed in lim2 cell lines and tumor tissues were eventually selected (Fig. 2D). Thereafter, the expression of five high-ranking candidate genes in lim2 cell lines was reconfirmed and three reliable candidates (ENST00000521989, ENST00000438897, and ENST00000507803) were eventually determined (Fig. 2E). RT-PCR experiments were performed on six paired tumor samples containing normal tissue, primary tumor, and liver metastasis to further detect lncRNA expression in GBC tissues. The results showed that lncBCL2L11 (ENST00000438897) and lncHMGB (ENST00000507803) were highly expressed in liver metastasis, whereas LINC01605 (ENST00000521989) expression showed no significant difference among the tissues (Fig. 2F).

### lncBCL2L11 promotes GBC cell metastasis by reducing lipid metabolism in vitro and in vivo

**Fig. 3:**
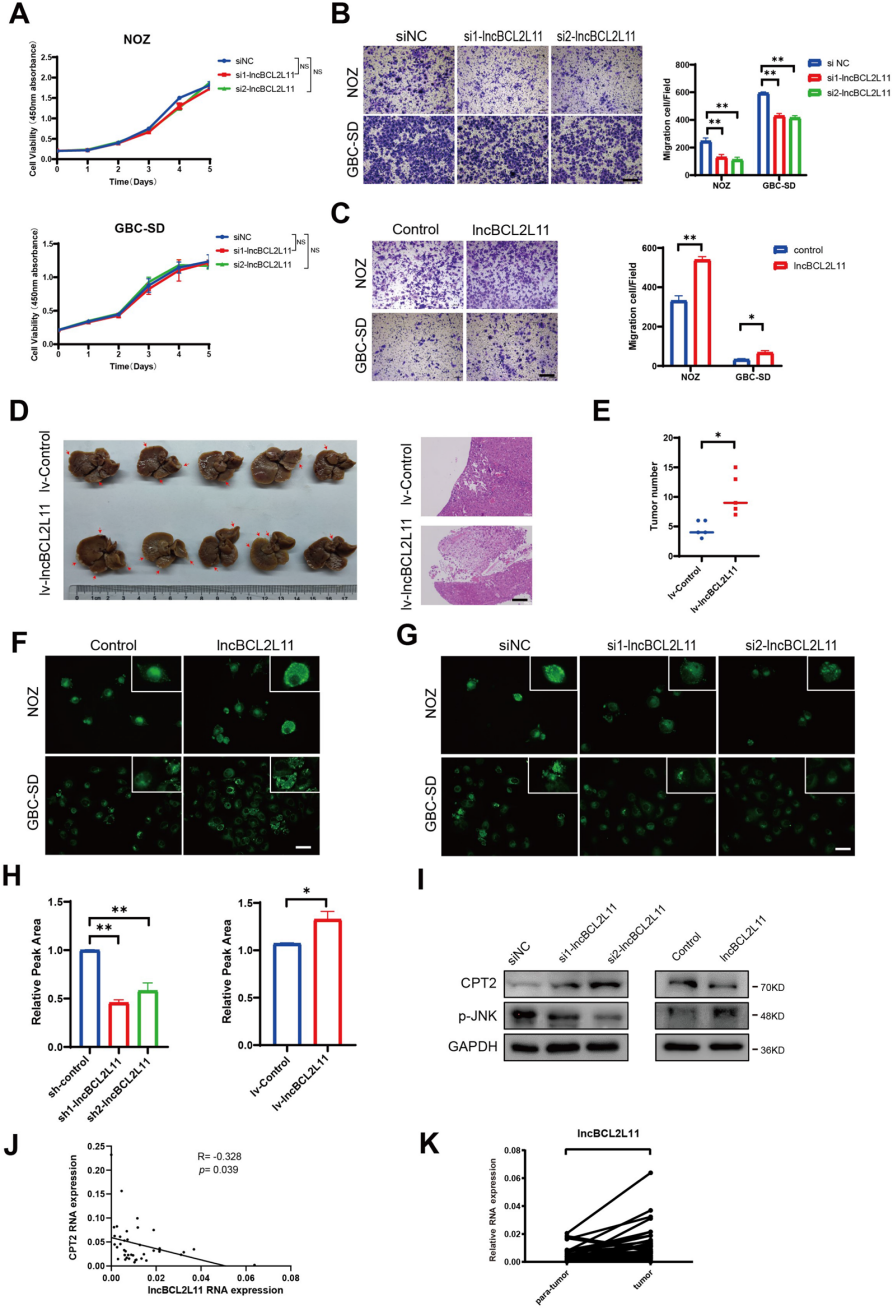
lncBCL2L11 promotes GBC cell metastasis by reducing lipid metabolism in vitro and in vivo. **A** Cell proliferation assay for NOZ and GBC-SD cells transfected with siRNAs were determined using CCK-8 assay. **B**, **C** Transwell assays were performed in NOZ and GBC-SD cells. Scale bars, 100 μm. **D** Liver metastasis in mice at 6 weeks after intrasplenic injection with NOZ, red arrows indicate metastatic nodules(left). Representative isolated liver tissue sections were stained by hematoxylin–eosin(right). Scale bars, 100 μm. **E** Tumor number of liver metastases was measured in mice after lncBCL2L11 lentivirus transfected. **F**, **G** Lipid droplets in NOZ and GBC-SD cells after lncBCL2L11 overexpressing and knockdown were stained by BODYPI. **H** Total acylcarnitines in indicated cells detected by LS/MS. **I** Protein levels of CPT2, JNK, p-JNK in NOZ cells after overexpressing or knockdown lncBCL2L11. **J** The correlation between the expression of lncBCL2L11 and CPT2 was determined through a linear regression analysis. Paired t test. **K** Expression of lncBCL2L11 was detected via Real-time PCR in 40 paired GBC tissues

We aimed to confirm the proliferative and migratory functions of the three selected lncRNAs. In loss-of-function assays, relevant small interfering RNAs (siRNAs) were transfected into cells. The results showed that LINC01605 and lncBCL2L11 knockdown did not affect cell proliferation over a 5-day culture period. However, a reduction in lncHMGB increased the proliferation of GBC-SD cells, but did not influence NOZ cells (Fig. 3A and Additional file 2: Fig. S3A). The transwell assay showed that only a decrease in lncBCL2L11 could downregulate cell migration in both cell lines (Fig. 3B and Additional file 2: Fig. S3B). Therefore, lncBCL2L11 was given continuous focus in subsequent research as it was the only gene among the three candidates that affected cell migration.

Next, the rapid amplification of the 5′ and 3′ cDNA ends assay was used to ensure the total length of 631 bp of lncBCL2L11 in NOZ cells (Fig.S3C). Sequence analysis of the open reading frame of lncBCL2L11 using the National Center for Biotechnology Information failed to predict a protein containing more than 50 amino acids (Additional file 2: Fig. S4A). The coding potential assessment tool also revealed that the coding probability of lncBCL2L11 was at a low level (0.0086) (Additional file 2: Fig. S4B), indicating that lncBCL2L11 is a non-protein-coding RNA [27].

After confirming the characteristics of lncBCL2L11, gain-of-function assays were conducted to determine the function of lncBCL2L11. Consistent with the results of loss-of-function assays, lncBCL2L11 overexpression increased the cell migration (Fig. 3C). For in vivo analyses, a tumor metastasis model was established by intrasplenic injection of tumor cells. NOZ cells stably overexpressing lncBCL2L11 displayed increased liver metastasis compared to the control group (Fig. 3D, E). Taken together, these data strongly support the hypothesis that lncBCL2L11 promotes GBC metastasis.

As lncBCL2L11 was identified from the sequencing data of cells with high metastatic capacity and tissues with low FAO metabolism, we investigated whether the alteration of lncBCL2L11 causes changes in lipids. Boron dipyrromethene(BODIPY) staining and liquid chromatography-mass spectrometry (LC/MS) analysis showed that lncBCL2L11 knockdown decreased the accumulation of lipids droplets and the content of acylcarnitines, and lncBCL2L11 overexpression induced the accumulation of lipids droplets and acylcarnitines (Fig. 3F, G and H). In addition, Western blot assay further revealed the upregulation of CPT2 and inhibition of p-JNK caused by a decrease in lncBCL2L11 (Fig. 3I). Which may account for lncBCL2L11 regulation on acylcarnitines.

We next analyzed the correlation between lncBCL2L11 and CPT2 in paired tumor tissues. The results showed that lncBCL2L11 was negatively correlated with CPT2 (Fig. 3J) and lncBCL2L11 levels were significantly higher in tumor tissues than in paracancerous tissues (Fig. 3K).

### Acylcarnitines stabilize lncBCL2L11 expression via m6A methylation

**Fig. 4:**
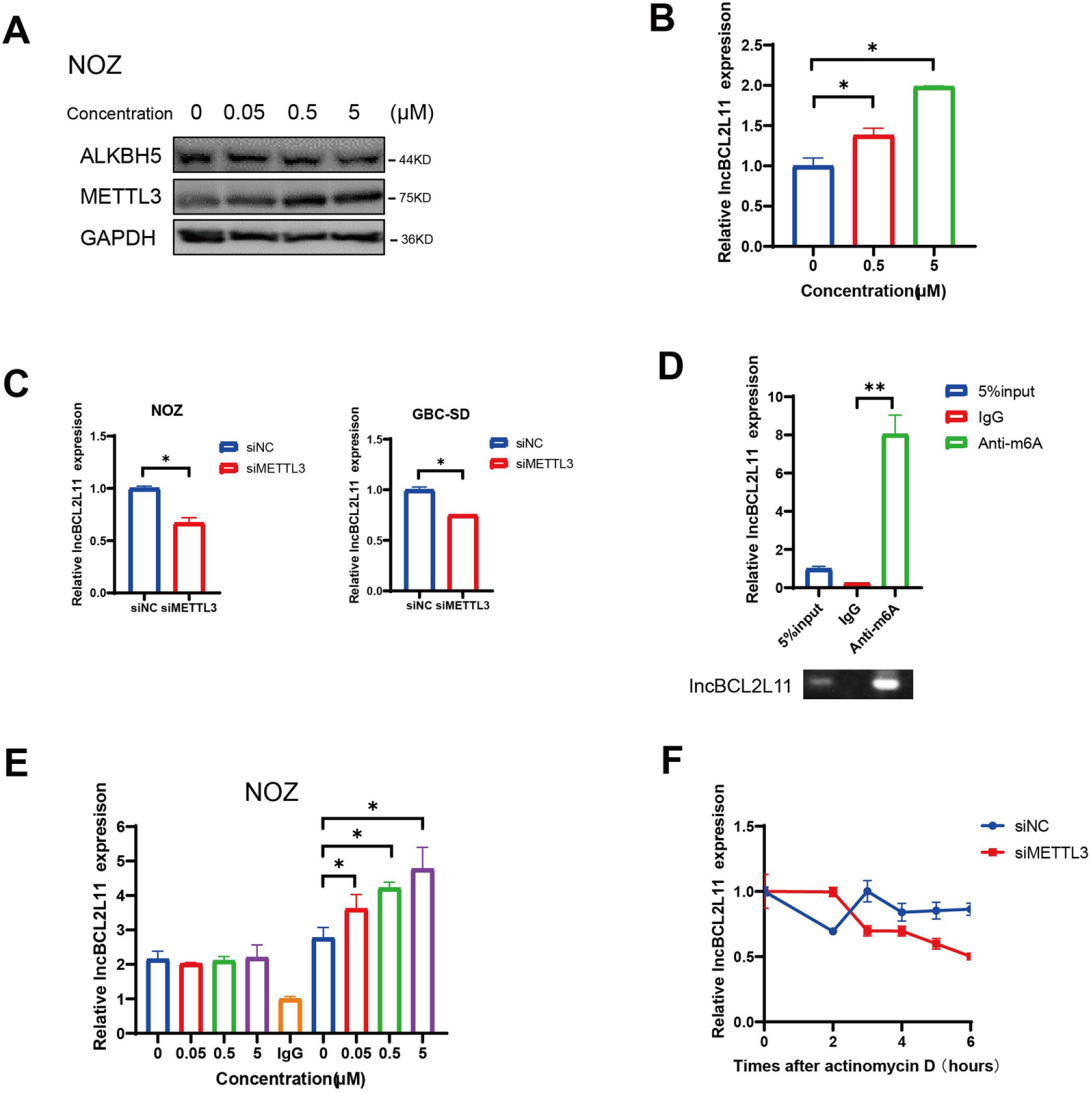
N6-methyladenosine modified is associated with the expression of lncBCL2L11. **A** Protein levels of ALKBH5 and METTL3 in NOZ cells cocultured with different concentrations of acylcarnitines. **B** Expression of lncBCL2L11 in NOZ cells co-cultured with acylcarnitine was detected by RT-PCR. **C** Expression of lncBCL2L11 was tested after METTL3 knockdown in NOZ and GBC-SD cells. **D** RIP assay was performed by anti-m6A antibody. LncBCL2L11 was detected by RT-PCR. **E** Methylation enrichment of lncBCL2L11 co-cultured with acylcarnitines was detected by m6A RIP. **F** Expression of lncBCL2L11 was detected by RT-PCR after actinomycin D inhibition (*p*=0.007)

Mutual regulation between lipid metabolism and m6A methylation has been reported [28, 29]. Disordered m6A modification can induce adipogenesis and cause the progress of non-alcoholic fatty liver disease and hepatocellular carcinoma [30]. In contrast, a high-fat diet also can induce m6A modification of lipid metabolism-related genes, including FAO and triglyceride metabolism-related genes [31]. Based on these reports, we hypothesized that the accumulation of acylcarnitines could alter m6A methylation in GBC cells. Tumor cells were co-cultured with different concentrations of acylcarnitines to test this hypothesis. Western blotting revealed that acylcarnitines upregulated m6A methylation levels in a dose-dependent manner in NOZ cells, including a decrease in the levels of the m6A demethylase ALKBH5 and an increase in the levels of the methylase METTL3 (Fig. 4A). Along with the methylation changes, lncBCL2L11 levels were also upregulated by incubation with acylcarnitines (Fig. 4B). Moreover, we found that METTL3 knockdown caused a decrease in lncBCL2L11, suggesting a potential regulation between lncBCL2L11 and m6A methylation (Fig. 4C).

Using SRAMP [32], an online m6A predictor, four highly probable m6A methylation motifs were identified in lncBCL2L11 (Additional file 2: Fig. S4C). Methylated RNA immunoprecipitation analysis revealed that m6A methylation was approximately eightfold in lncBCL2L11 compared to the control group (Fig. 4D), and m6A methylation enrichment of lncBCL2L11 showed a dose-dependent pattern with acylcarnitines (Fig. 4E). To detect the influence of methylation on RNA expression, RNA transcription was inhibited with cycloheximide, and the degradation rate of lncBCL2L11 was detected. QRT-PCR showed that METTL3 suppression accelerated the half-life of lncBCL2L11 in GBC cells (Fig. 4F). These data support the hypothesis that acylcarnitines can stabilize lncBCL2L11 expression through m6A methylation.

### lncBCL2L11 interacts with THOC6 and regulates THOC5 stability

**Fig. 5:**
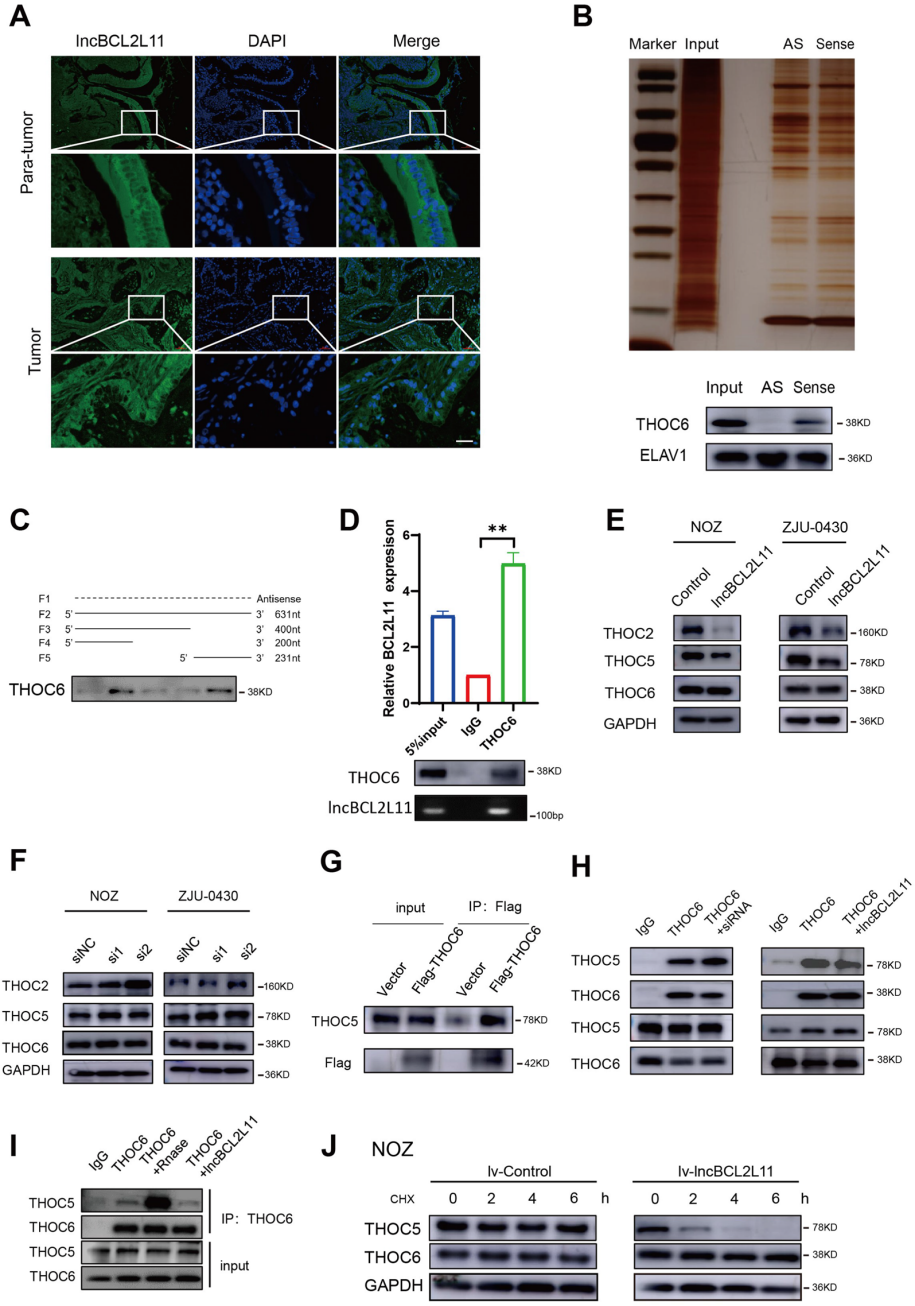
lncBCL2L11 interacts with THOC6 and regulates THOC5 stability. **A** FISH assay was performed to exam the location of lncBCL2L11 in GBC tissues. Scale bar, 100 μm. **B** RNA pull down assay by lncBCL2L11 and its antisense RNA followed by silver staining. Differential protein band was excised for mass spectrometry analysis. Western blot analysis revealed the specific binding between lncBCL2L11 and THOC6.ELAV1 used as negative control. **C** Immunoblotting of THOC6 in pull-down samples by biotin labeled anti-sense lncBCL2L11 (F1), full-length lncBCL2L11 (F2) and truncated lncBCL2L11 framents (F3:1-400nt; F4:1-200nt; F5:400-631nt). **D** RIP assay was performed using anti-THOC6 antibody in NOZ extracts followed by RT-PCR. **E**, **F** Protein levels of THOC2, THOC5, THOC6 after lncBCL2L11 overexpression and knockdown. **G** Interaction between THOC5 and THOC6 was detected by CO-IP assay. **H**, **I** CO-IP assay was performed to detected the interaction between THOC6 and THOC5 after endogenous or exogenous increase or decrease of lncBCL2L11. **J** Western blot of proteins from control and lncBCL2L11 overexpression NOZ cells after adding actinomycin to prevent protein synthesis

Spatial localization of lncRNAs affects their function [33, 34]; therefore, cellular localization of lncBCL2L11 in GBC cells was first detected. RNAs in the cytoplasm and nucleus of NOZ and GBC-SD cells were isolated and detected using qRT-PCR. The results revealed that lncBCL2L11 was mainly located in the nucleus (Additional file 2: Fig. S5A), consistent with fluorescence in situ hybridization assay results (Additional file 2: Fig. S5B). Moreover, fluorescence in situ hybridization experiments were also performed on para-tumor epithelial cells and cancer cells in paired gallbladder tissues. The results showed that lncBCL2L11 was still mainly located in the nucleus of the tumor cells, whereas it was mostly concentrated in the cytoplasm in normal gallbladder epithelial cells (Fig. 5A). Although the mechanism underlying the difference in the location of lncBCL2L11 is unclear, we can infer that lncBCL2L11 mainly functions as a critical oncogene in the nucleus.

The interaction between lncRNAs and proteins is one of the main molecular mechanisms of lncRNA [35, 36]. To identify the downstream regulatory mechanism of lncBCL2L11 in GBC cells, a biotin-labeled RNA pulldown assay and LC/MS analysis were performed. Using silver staining, we identified a specific novel band at 36 KDa (which could be the interacting protein of lncBCL2L11) compared to the control group. Next, a series of candidate nuclear proteins with molecular weights ranging from 35 to 40 KDa were analyzed and screened (Additional file 2: Table S2). The specific interaction protein THOC6 was finally verified using western blotting (Fig. 5B). To further determine the interaction between the two molecules and their binding regions, three fragments of lncBCL2L11 (1–200 bp, 1–400 bp, 400-631 bp) and biotinylated RNA were generated via in vitro transcription. An RNA pulldown assay performed in NOZ lysate showed that the 3′ fragment of lncBCL2L11 mediated the interaction with THOC6 (Fig. 5C). Concurrently, a THOC6 antibody was used to immunoprecipitate endogenous THOC6 protein from cell lysates, and THOC6-bound RNA was extracted. RT-PCR data revealed a ~ fivefold enrichment of lncBCL2L11 in the anti-THOC6 group compared to the control group (Fig. 5D), which suggested direct binding of lncBCL2L11 to THOC6 in GBC.

THOC6 may be involved in mRNA transcription, processing, and nuclear transport [37]. Therefore, we first examined whether the interaction of lncBCL2L11 and THOC6 resulted in changes in their expression or location. Immunofluorescence (IF) and RNA isolation results showed that neither knockdown nor overexpression of lncBCL2L11 or THOC6 altered the expression and localization of the other (Additional file 2: Fig. S5D–F and Fig. S6A, B). As THOC6 usually functions in the THO complex, we further speculated that lncBCL2L11 could affect the formation of THO complex, similar to THOC2, which functions as a scaffold, and THOC5, which acts as a major functional protein in the complex. The reasons supporting our hypothesis are as follows: RNA pulldown assay confirmed the interaction between lncBCL2L11 and THOC6 (Fig. 5C) in tumor cells. Western blotting revealed that lncBCL2L11 overexpression decreased the expression of THOC2 and THOC5, and lncBCL2L11 knockdown upregulated THOC2 and THOC5 (Fig. 5E, F); co-immunoprecipitation (CO-IP) assays confirmed the direct combination of THOC5 and THOC6 in GBC cells (Fig. 5G). These data implied that lncBCL2L11 could competitively impair the binding of THOC5 and THOC6. To further confirm this conjecture, CO-IP assay was performed after knockdown or overexpression of lncBCL2L11 in NOZ cells. Western blotting showed enhanced interaction between THOC5 and THOC6 in lncBCL2L11 suppressing cells and reduced interaction in lncBCL2L11 overexpressing cells (Fig. 5H). Consistent with this result, exogenous reduction or increase in lncBCL2L11 in the cell lysate followed by Co-IP assay indicated that the binding of THOC6 and THOC5 is regulated by lncBCL2L11 (Fig. 5I). Then, protein synthesis was inhibited by cycloheximide and the degradation of the THO complex was analyzed. Predictably, an increase in lncBCL2L11 accelerated the half-life of THOC5, but had little effect on THOC6 (Fig. 5J and Additional file 2: Fig. S6C). These data were consistent with the hypothesis that enhanced lncBCL2L11 expression could inhibit THO complex formation and result in the accelerated degradation of free THOC5.

### THOC5 is involved in the biological function of lncBCL2L11

**Fig. 6:**
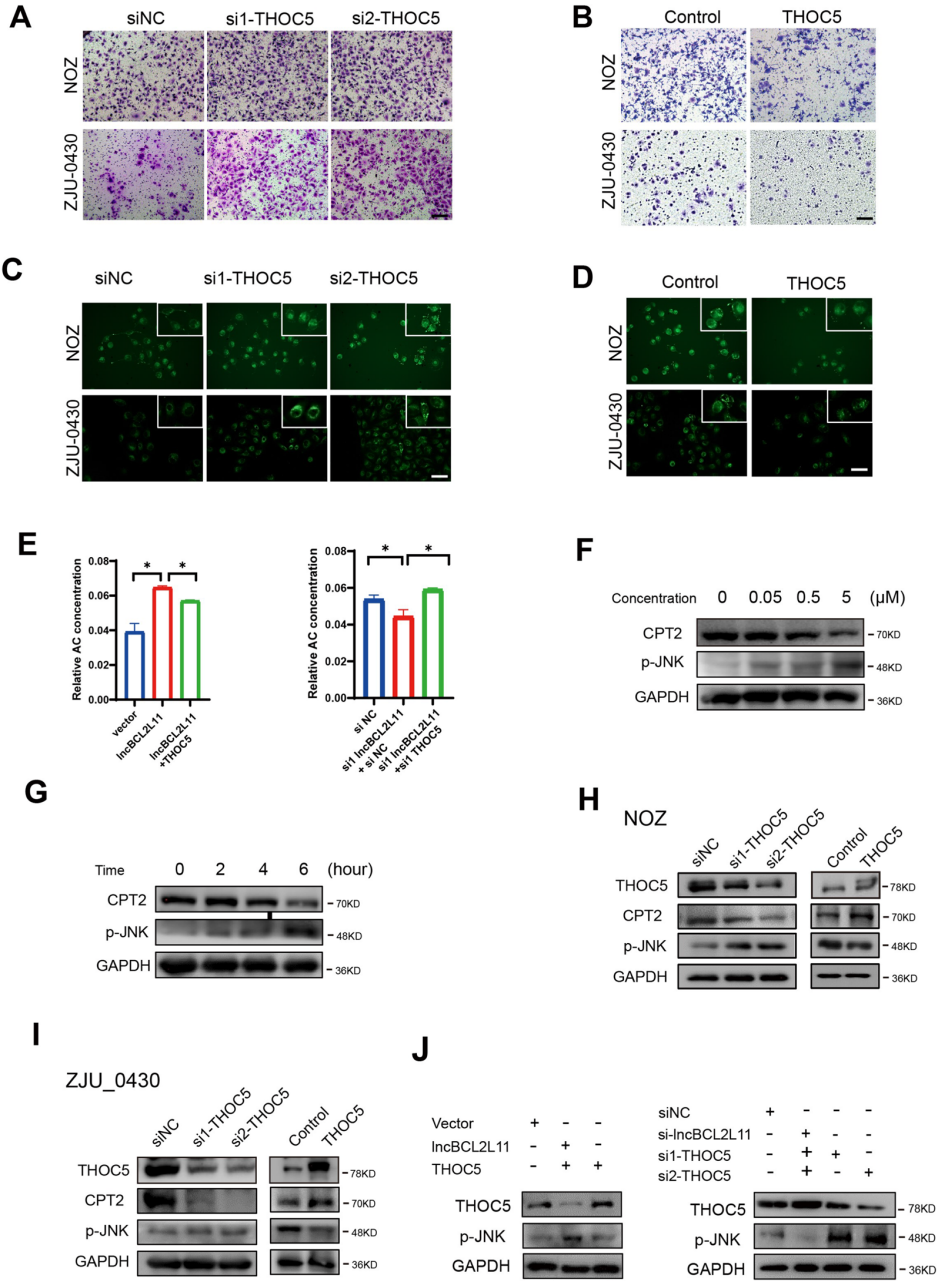
THOC5 promotes gallbladder cancer metastasis through lipid metabolism. **A**, **B** Transwell assays of NOZ and ZJU-0430 cells transfected with siRNAs and plasmids. Scale bars, 100 μm. **C**, **D** Lipid droplets in NOZ cells after THOC5 overexpressing and knockdown were stained by BODYPI. **E** Total acylcarnitine in NOZ cells in indicated cells. **F** Protein levels of CPT2 and p-JNK were determined in NOZ cells after incubating with different concentrations of acylcarnitine for 6 h. **G** Protein levels of CPT2 and p-JNK in NOZ cells after incubating with 5 μM acylcarnitine for different hours. **H**, **I** Protein levels of CPT2 and p-JNK in GBC cells after THOC5 knockdown or overexpressing. **J** Protein levels of CPT2 and p-JNK in NOZ cells in indicated cells

We next examined the potential mechanism of THOC5 in GBC metastasis. Migration assay showed that THOC5 knockdown strongly promoted cell metastasis and THOC5 overexpression dramatically inhibited the migration of NOZ and ZJU-0430 cells (Fig. 6A). As THOC5 has been reported to be associated with lipid accumulation in hepatocellular carcinoma cells, it is reasonable to postulate that THOC5 participates in the biological regulation of lncBCL2L11 in GBC. To clarify this signaling in our system, we examined the lipid content in GBC cells via BODIPY staining. The results showed that THOC5 knockdown caused the accumulation of neutral lipid droplets, whereas THOC5 overexpression reduced lipid levels in GBC cells (Fig. 6C, D). LC/MS results showed the introduction of THOC5 into lncBCL2L11-overexpressed cells decreased the content of acylcarnitines and the inhibition of THOC5 partially restored the levels inhibited by lncBCL2L11 siRNA (Fig. 6E and Additional file 2: Fig. S6D). The accumulated acylcarnitines activated JNK phosphorylation and inhibits the expression of CPT2 in a dose and time-dependent manner (Fig. 6F, G). We further confirmed the influence of THOC5 on p-JNK and CPT2 through Western blot assay. The results showed a decrease in p-JNK expression and an increase in CPT2 in THOC5-overexpressed cells and a decrease in CPT2 expression and an increase in p-JNK expression in THOC5-knockdown cells (Fig. 6H, I). In addition, THOC5 also partially restored the effect of lncBCL2L11 on CPT2 and p-JNK protein levels (Fig. 6J). These data indicate that THOC5 may upregulate acylcarnitines and activate JNK phosphorylation, thereby promoting cell migration in GBC.

**Fig. 7:**
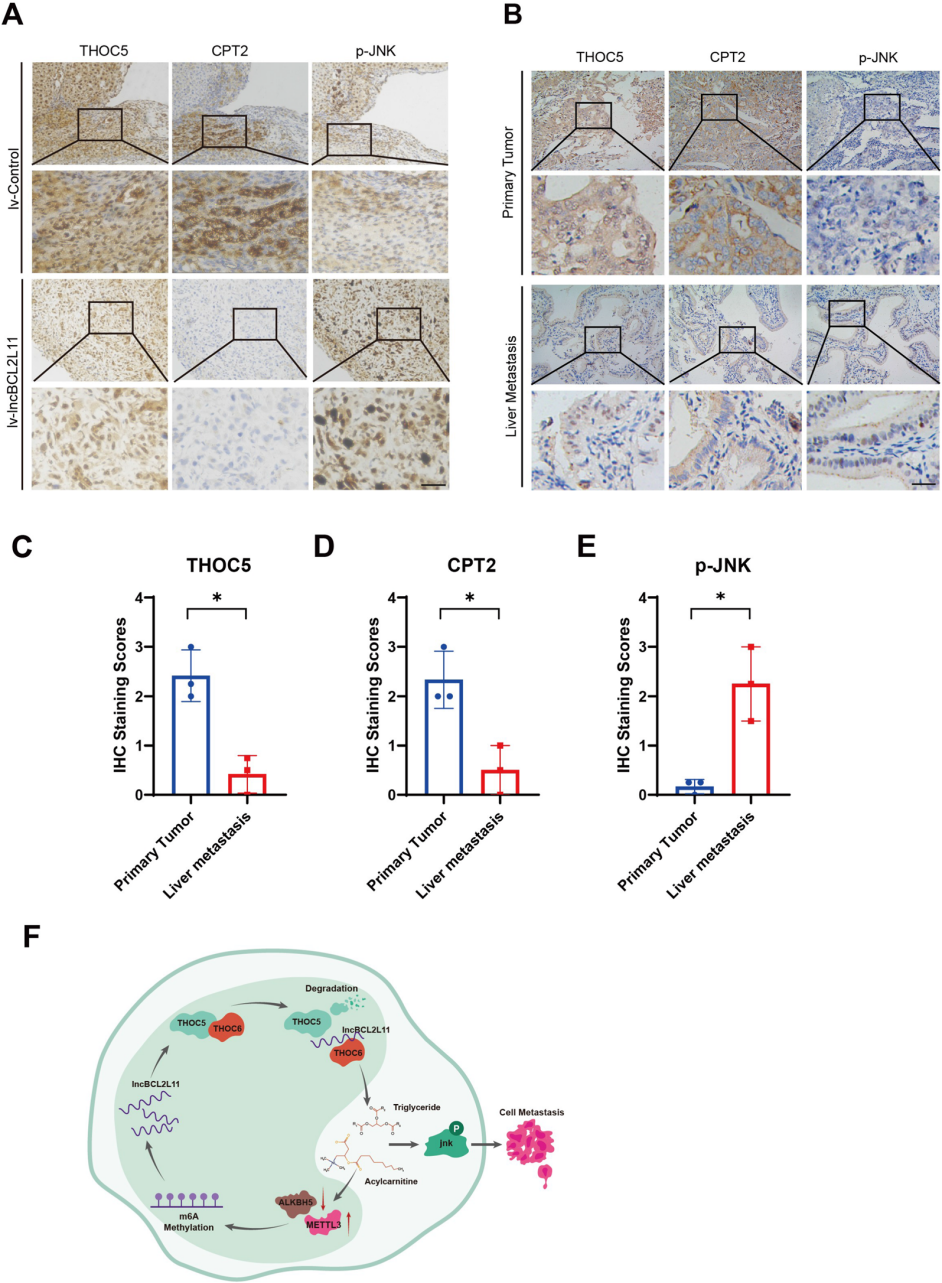
Expression of related proteins in GBC. **A** Representative images of THOC5, CPT2, p-JNK by immunohistochemical staining from control and lncBCL2L11-overexpressing liver metastases. Scale bars, 100 μm. **B** Representative images of THOC5, CPT2, p-JNK in primary tumor and liver metastases. Scale bars, 100 μm. **C**–**E** Scatterplots of the average IHC staining scores of related proteins. **F** Functional diagram of lncBCL2L11 in promoting metastasis in GBC Cells

Finally, we performed immunohistochemical staining to confirm the expression of different targets in tumor samples. Both lncBCL2L11-overexpressing tumors and metastatic samples displayed decreased expression of THOC5 and CPT2 and increased JNK phosphorylation (Fig. 7A–E and Additional file 2: Fig. S7A). These observations indicate that the regulation of THOC5 is required for lncBCL2L11 to exert biological functions in GBC.

## Discussion

Tumor progression is accompanied by abnormal lipid metabolism. There are three reasons for focusing on lipid metabolism in GBC. (1) Bile in the gallbladder is an important participant in lipid metabolism. The occurrence of tumor may lead to alterations in the quantity and quality of the bile, causing abnormal lipid metabolism. (2) Anatomically, the gallbladder is located near the liver which is the most important lipid metabolism organ. High lipids are one of the main microenvironments associated with GBC. (3) Obesity is a key risk factor for GBC [38–40].

Previous studies attempted to identify a diagnostic lipid marker in GBC, including elevated phospholipids, Cholesrerol esters and plasmalogen and depleted TG [20]. Other studies indicated that changes in fatty acid synthase (FASN) promotes gallbladder cancer progression through PI3K/AKT signaling [21]. It remains unclear whether lipids are byproducts of tumor driving genes or direct factors in tumorgenesis. In this study, the malignant effects of TG and acylcarnitines were identified. However, TG shows a decreasing trend in tumor tissue, which seems to contradict its biological effects. We consider these changes may be due to the rapid energy consumption of tumor cells and enhancement of cell membrane synthesis. Serum TG may more accurately reflects the current status of patients. Clinical data also indicates that high serum TG was associated with liver metastasis and poorer survival in GBC patients. Our findings to some extent explain the epidemiological research findings that obesity is a high-risk factor for GBC. In the current situation where obesity has become a global issue, this may have important implications for the prevention and treatment of tumors. Reducing the intake of TGs may be an effective and practical method to improve the prognosis of GBC.

As a novel finding in this study, acylcarnitines were found to be elevated in tumor tissues of GBC patients. Given the migration promoting function of acylcarnitines, by joint analysis of RNA sequencing data from GBC tumor tissues and highly metastatic GBC cells, we identified a migration related gene lncBCL2L11. LncRNA has been shown to participate in cancer progression through various metabolic pathways. Here, we report that lncBCL2L11 directly binds to THOC6 to inhibit the formation of the THO complex, leading to lipid accumulation and further promoting malignant progression of GBC. Indeed, such feedback loops are frequent in cancer. For example, lncRNA ENO1-it1 regulating glycolysis through KAT7 to promote malignant transformation of colorectal cancer [41]. Unfortunately, the degradation pathway of THOC5, the binding partner of THOC6, is still unknown and needs further investigation.

N6 methyladenosine (m6A) is the most abundant epigenetic modification of eukaryotic messenger RNA (mRNA). Regulation of m6A mRNA expression plays an important role in adipogenesis [42], liver lipid metabolism [43], obesity [44] and other metabolic diseases. After HFD feeding, m6A methylation level and major m6A methyltransferase Mettl3 consistently elevated in the liver of mouse [43, 45]. This is highly consistent with our conclusion that when gallbladder cancer cells consume excessive exogenous lipids, the RNA methylation level and METTL3 continue to upregulate. The reason for METTL3 increase was considered as a result of FTO-induced decrease in m6A or maybe result from other signal pathways [45]. Here, we confirmed this hypothesis that intake of acylcarnitines affects m6A modification and predicted the possible methylation sites of lncBCL2L11. However, the exact physical interaction remains to be established via biologically engineering approaches.

It is known that the JNK pathway is involved in inflammation, cell apoptosis, cell differentiation, and proliferation. Recently, multiple studies have reported the key role of JNK in tumor regulation. JNK is considered as a potential target for cancer treatment. In GBC, JNK may promote the progression of tumor cells through the JNK/p38 MAPK [46] or JNK c-Jun pathway [47]. Here, we found that acylcarnitine contributes to the activation of JNK phosphorylation. This finding will enrich our understanding of JNK signaling and is worth further investigating whether acylcarnitine is a true component of JNK signaling complexes.

Undoubtedly, there are some limitations in our research. When discussing the clinical significance of high serum triglycerides in tumor invasion and metastasis, a retrospective study was chosen. Although this contains a large number of samples, the power of the argument is limited, and a further randomized controlled trial is needed. In addition, there may be a bias in the selection of research subjects due to the diversity of lipid molecules, and we can never rule out the existence of lipid molecules with different functions. Systematic lipid function screening to identify dysfunctional lipid molecules may potentially address the aforementioned issue. Furthermore, utilizing biotechnological techniques such as CRISPR screening to explore the direct action targets of lipid molecules holds significant importance for the treatment of GBC patients.

## Conclusions

In this study, we found that acylcarnitines can promote gallbladder cancer metastasis through lncBCL2L11-THOC5-JNK axis. Given the clinical significance and biochemical function of lncBCL2L11 in GBC, we conclude that lncBCL2L11 and its related pathways are critical for lipid regulation and GBC progression. A low-fat diet or treatment targeting this pathway is of great significance for preventing and treating GBC.

### Supplementary Information

**Additional file 1: Table S1.** Lipid composition of 5 paired GBC tissues. **Table S2.** Primary antibodies used in this study. **Table S3.** The potential lncBCL2L11-interacting proteins identified by mass spectrometry. **Table S4.** Primer and Probe Sequences Used in This Study. **Table S5.** siRNA and shRNA sequences used and lncBCL2L11 probe for FISH in This Study.

**Additional file 2:** **Figure S1.** **A** Principal Component Analysis (PCA) of the lipomics in 5 paired GBC tissues. **B** Hierarchical clustering analysis of the lipomics in 5 paied GBC tissues. **C**, **D** Expression of different types of triglycerides and fatty acids in gallbladder carcinoma compared to adjacent tissues. **Figure S2**. **A** GO enrichment analysis of differential genes in GBC tissues. **B** FAO related genes in GSE139683. **C** RNA expression levels of FAO metabolism related genes LIPE, ACSL4, PNPLA2, CRAT, CT2, OCTN2. **D** The image of liver metastasis in mice with NOZ cells injected into spleen after 4 weeks of high-fat diet. **Figure S3. A** Cell proliferation was detected after knockdown of LINC01605 and HMGB2 by CCK-8 assay. **B** Detection of cell migration after knockdown of LINC01605 and HMGB2 by transwell assay. **C** Sequence identification of full length lncBCL2L11 in NOZ cells through 5′ and 3′ rapid amplification of cDNA ends (RACE) assays. **Figure S4. A** Prediction of potential encoding proteins of lncBCL2L11 by ORF finder. **B** Coding potential of lncBCL2L11 predicted by CPAT. **C** Prediction of m6A methylation sites in lncBCL2L11 using SRAMP. **Figure S5. A** Localization of lncBCl2L11 in NOZ cells and GCB-SD cells by RT-PCR after Isolation of cytoplasmic and nuclear RNA, MALAT1 served as a reference of cytoplasm and MTND, HOTAIR served as a reference of nuclear. **B** Fluorescence in Situ Hybridization (FISH) assay (green) used to examine the expression and location of lncBCL2L11 in NOZ and GBC-SD cells. **C** Identification of biotin-labeled lncBCL2L11 synthesized in vitro. **D** Localization of THOC6 in lncBCL2L11-overexpressing GBC-SD cells by Immunofluorescence assay. **E** Protein detection of THOC6 in isolated cytoplasmic and nuclear protein of lncBCL2L11-overexpressing GBC-SD cells. Tubulin was used as the internal reference of cytoplasm and Histone H3 as the internal reference of nucleus. **F** Detection of lncBCL2L11 in nucleus and cytoplasm of THOC6 knockdown NOZ cells by RT-PCR. **Figure S6. A**, **B** Alteration of lncBCL2L11 expression after knockdown of THOC6 in NOZ and GBC-SD cells. **C** Degradation rate of THOC5 and THOC6 in lncBCL2L11-overexpressing ZJU-0430 cells. **D** Detection of different acylcarnitine concentrations in indicated cells. **Figure S7. A** Representative images of THOC5, CPT2, p-JNK in primary tumor and liver metastases. Scale bars, 100 μm.

## Data Availability

The datasets used and/or analyzed during the current study are available from the corresponding author on reasonable request.

## Abbreviations

Co-IP: Coimmunoprecipitation
FAO: Fatty acid β-oxidation
GBC: Gallbladder cancer
LC/MS: Liquid chromatography-mass spectrometry
lncRNA: Long non-coding RNA
m6A: N^6^-methyladenosine
MS: Mass spectrometry
qRT-PCR: Quantitative real-time PCR
siRNA: Small interfering RNA
RACE: Rapid amplification of the cDNA ends
MeRIP: Methylated RNA Immunoprecipitation
RIP: RNA immunoprecipitation
FISH: Fluorescence in situ hybridization
IHC: Immunohistochemistry
KEGG: Gene Ontology and Kyoto Encyclopedia of Genes and Genomes

## References

[1] Wu XS, Shi LB, Li ML (2014). Evaluation of two inflammation-based prognostic scores in patients with resectable gallbladder carcinoma. Ann Surg Oncol.

[2] Butte JM, Matsuo K, GöNEN M (2011). Gallbladder cancer: differences in presentation, surgical treatment, and survival in patients treated at centers in three countries. J Am Coll Surg.

[3] Xiang S, Wang Z, Ye Y (2019). E2F1 and E2F7 differentially regulate KPNA2 to promote the development of gallbladder cancer. Oncogene.

[4] Zhang L, Jiang L, Zeng L (2023). The oncogenic role of NF1 in gallbladder cancer through regulation of YAP1 stability by direct interaction with YAP1. J Transl Med.

[5] Zhao C, Yang ZY, Zhang J (2022). Inhibition of XPO1 with KPT-330 induces autophagy-dependent apoptosis in gallbladder cancer by activating the p53/mTOR pathway. J Transl Med.

[6] Baiu I, Visser B (2018). Gallbladder cancer. JAMA.

[7] Song X, Hu Y, Li Y (2020). Overview of current targeted therapy in gallbladder cancer. Signal Transduct Target Ther.

[8] Azizi AA, Lamarca A, McNamara MG (2021). Chemotherapy for advanced gallbladder cancer (GBC): a systematic review and meta-analysis. Crit Rev Oncol Hematol.

[9] Squadroni M, Tondulli L, Gatta G (2017). Cholangiocarcinoma. Crit Rev Oncol Hematol.

[10] Shih SP, Schulick RD, Cameron JL (2007). Gallbladder cancer: the role of laparoscopy and radical resection. Ann Surg.

[11] Pawlik TM, Gleisner AL, Vigano L (2007). Incidence of finding residual disease for incidental gallbladder carcinoma: implications for re-resection. J Gastrointest Surg.

[12] Vander Heiden MG, Deberardinis RJ (2017). Understanding the intersections between metabolism and cancer biology. Cell..

[13] Vasseur S, Guillaumond F (2022). Lipids in cancer: a global view of the contribution of lipid pathways to metastatic formation and treatment resistance. Oncogenesis.

[14] Qu Q, Zeng F, Liu X (2016). Fatty acid oxidation and carnitine palmitoyltransferase I: emerging therapeutic targets in cancer. Cell Death Dis.

[15] de Oliveira MP, Liesa M (2020). The role of mitochondrial fat oxidation in cancer cell proliferation and survival. Cells.

[16] Ma Y, Temkin SM, Hawkridge AM (2018). Fatty acid oxidation: an emerging facet of metabolic transformation in cancer. Cancer Lett.

[17] Attané C, Muller C (2020). Drilling for oil: tumor-surrounding adipocytes fueling cancer. Trends Cancer..

[18] Wu H, Liu B, Chen Z (2020). MSC-induced lncRNA HCP5 drove fatty acid oxidation through miR-3619-5p/AMPK/PGC1α/CEBPB axis to promote stemness and chemo-resistance of gastric cancer. Cell Death Dis.

[19] Han J, Qu H, Han M (2021). MSC-induced lncRNA AGAP2-AS1 promotes stemness and trastuzumab resistance through regulating CPT1 expression and fatty acid oxidation in breast cancer. Oncogene.

[20] Jayalakshmi K, Sonkar K, Behari A (2011). Lipid profiling of cancerous and benign gallbladder tissues by 1H NMR spectroscopy. NMR Biomed.

[21] Cheng H, Sun Y, Yu X (2023). FASN promotes gallbladder cancer progression and reduces cancer cell sensitivity to gemcitabine through PI3K/AKT signaling. Drug Discov Ther.

[22] Zhang Y, Liu Y, Duan J (2019). Cholesterol depletion sensitizes gallbladder cancer to cisplatin by impairing DNA damage response. Cell Cycle (Georgetown, Tex).

[23] Tsugawa H, Cajka T, Kind T (2015). MS-DIAL: data-independent MS/MS deconvolution for comprehensive metabolome analysis. Nat Methods.

[24] Wu XS, Wang F, Li HF (2017). LncRNA-PAGBC acts as a microRNA sponge and promotes gallbladder tumorigenesis. EMBO Rep.

[25] Ren T, Li Y, Zhang X (2021). Protocol for a gallbladder cancer registry study in China: the Chinese Research Group of Gallbladder Cancer (CRGGC) study. BMJ Open.

[26] Li H, Hu Y, Jin Y (2020). Long noncoding RNA lncGALM increases risk of liver metastasis in gallbladder cancer through facilitating N-cadherin and IL-1β-dependent liver arrest and tumor extravasation. Clin Transl Med.

[27] Wang L, Park HJ, Dasari S (2013). CPAT: coding-potential assessment tool using an alignment-free logistic regression model. Nucleic Acids Res.

[28] Zhao Z, Meng J, Su R (2020). Epitranscriptomics in liver disease: basic concepts and therapeutic potential. J Hepatol.

[29] Yang Z, Yu GL, Zhu X (2022). Critical roles of FTO-mediated mRNA m6A demethylation in regulating adipogenesis and lipid metabolism: implications in lipid metabolic disorders. Genes Dis.

[30] Yang Y, Cai J, Yang X (2022). Dysregulated m6A modification promotes lipogenesis and development of non-alcoholic fatty liver disease and hepatocellular carcinoma. Mol Ther.

[31] Xu Z, Qin Y, Lv B (2022). Intermittent fasting improves high-fat diet-induced obesity cardiomyopathy via alleviating lipid deposition and apoptosis and decreasing m6a methylation in the heart. Nutrients.

[32] Zhou Y, Zeng P, Li YH (2016). SRAMP: prediction of mammalian N6-methyladenosine (m6A) sites based on sequence-derived features. Nucleic Acids Res.

[33] Bridges MC, Daulagala AC, Kourtidis A (2021). LNCcation: lncRNA localization and function. J Cell Biol.

[34] Statello L, Guo CJ, Chen LL (2021). Gene regulation by long non-coding RNAs and its biological functions. Nat Rev Mol Cell Biol.

[35] Tang J, Yan T, Bao Y (2019). LncRNA GLCC1 promotes colorectal carcinogenesis and glucose metabolism by stabilizing c-Myc. Nat Commun.

[36] Wu ZR, Yan L, Liu YT (2018). Inhibition of mTORC1 by lncRNA H19 via disrupting 4E-BP1/Raptor interaction in pituitary tumours. Nat Commun.

[37] Ruaud L, Roux N, Boutaud L (2022). Biallelic THOC6 pathogenic variants: prenatal phenotype and review of the literature. Birth defects Res.

[38] Parra-Landazury NM, Cordova-Gallardo J, MéNDEZ-SáNCHEZ N (2021). Obesity and gallstones. Visc Med.

[39] Wang F, Wang B, Qiao L (2012). Association between obesity and gallbladder cancer. Front Biosci.

[40] Sharma A, Sharma KL, Gupta A (2017). Gallbladder cancer epidemiology, pathogenesis and molecular genetics: recent update. World J Gastroenterol.

[41] Hong J, Guo F, Lu SY (2021). *F*. *nucleatum* targets lncRNA ENO1-IT1 to promote glycolysis and oncogenesis in colorectal cancer. Gut..

[42] Zhao X, Yang Y, Sun BF (2014). FTO-dependent demethylation of N6-methyladenosine regulates mRNA splicing and is required for adipogenesis. Cell Res.

[43] Zhong X, Yu J, Frazier K (2018). Circadian clock regulation of hepatic lipid metabolism by modulation of m(6)A mRNA methylation. Cell Rep.

[44] Lu N, Li X, Yu J (2018). Curcumin attenuates lipopolysaccharide-induced hepatic lipid metabolism disorder by modification of m(6) A RNA methylation in piglets. Lipids.

[45] Li Y, Zhang Q, Cui G (2020). m(6)A regulates liver metabolic disorders and hepatogenous diabetes. Genomics Proteomics Bioinformatics.

[46] Liu S, Chu B, Cai C (2020). DGCR5 promotes gallbladder cancer by sponging MiR-3619-5p via MEK/ERK1/2 and JNK/p38 MAPK pathways. J Cancer.

[47] Miao H, Geng Y, Li Y (2022). Novel protein kinase inhibitor TT-00420 inhibits gallbladder cancer by inhibiting JNK/JUN-mediated signaling pathway. Cell Oncol (Dordr).

