# Supplementary material for: Acylcarnitines promote gallbladder cancer metastasis through lncBCL2L11-THOC5-JNK axis
Source: J Transl Med. 2024 Mar 22;22:299. doi: 10.1186/s12967-024-05091-0 (PMC10958842; doi:10.1186/s12967-024-05091-0)
Supplement: Supplementary file 2 — Additional file 2: Figure S1. A Principal Component Analysis (PCA) of the lipomics in 5 paired GBC tissues. B Hierarchical clustering analysis of the lipomics in 5 paied GBC tissues. C, D Expression of different types of triglycerides and fatty acids in gallbladder carcinoma compared to adjacent tissues. Figure S2. A GO enrichment analysis of differential genes in GBC tissues. B FAO related genes in GSE139683. C RNA expression levels of FAO metabolism related genes LIPE, ACSL4, PNPLA2, CRAT, CT2, OCTN2. D The image of liver metastasis in mice with NOZ cells injected into spleen after 4 weeks of high-fat diet. Figure S3. A Cell proliferation was detected after knockdown of LINC01605 and HMGB2 by CCK-8 assay. B Detection of cell migration after knockdown of LINC01605 and HMGB2 by transwell assay. C Sequence identification of full length lncBCL2L11 in NOZ cells through 5′ and 3′ rapid amplification of cDNA ends (RACE) assays. Figure S4. A Prediction of potential encoding proteins of lncBCL2L11 by ORF finder. B Coding potential of lncBCL2L11 predicted by CPAT. C Prediction of m6A methylation sites in lncBCL2L11 using SRAMP. Figure S5. A Localization of lncBCl2L11 in NOZ cells and GCB-SD cells by RT-PCR after Isolation of cytoplasmic and nuclear RNA, MALAT1 served as a reference of cytoplasm and MTND, HOTAIR served as a reference of nuclear. B Fluorescence in Situ Hybridization (FISH) assay (green) used to examine the expression and location of lncBCL2L11 in NOZ and GBC-SD cells. C Identification of biotin-labeled lncBCL2L11 synthesized in vitro. D Localization of THOC6 in lncBCL2L11-overexpressing GBC-SD cells by Immunofluorescence assay. E Protein detection of THOC6 in isolated cytoplasmic and nuclear protein of lncBCL2L11-overexpressing GBC-SD cells. Tubulin was used as the internal reference of cytoplasm and Histone H3 as the internal reference of nucleus. F Detection of lncBCL2L11 in nucleus and cytoplasm of THOC6 knockdown NOZ cells by RT-PCR. Figure S6. A, B Alter [file 12967_2024_5091_MOESM2_ESM.pdf]

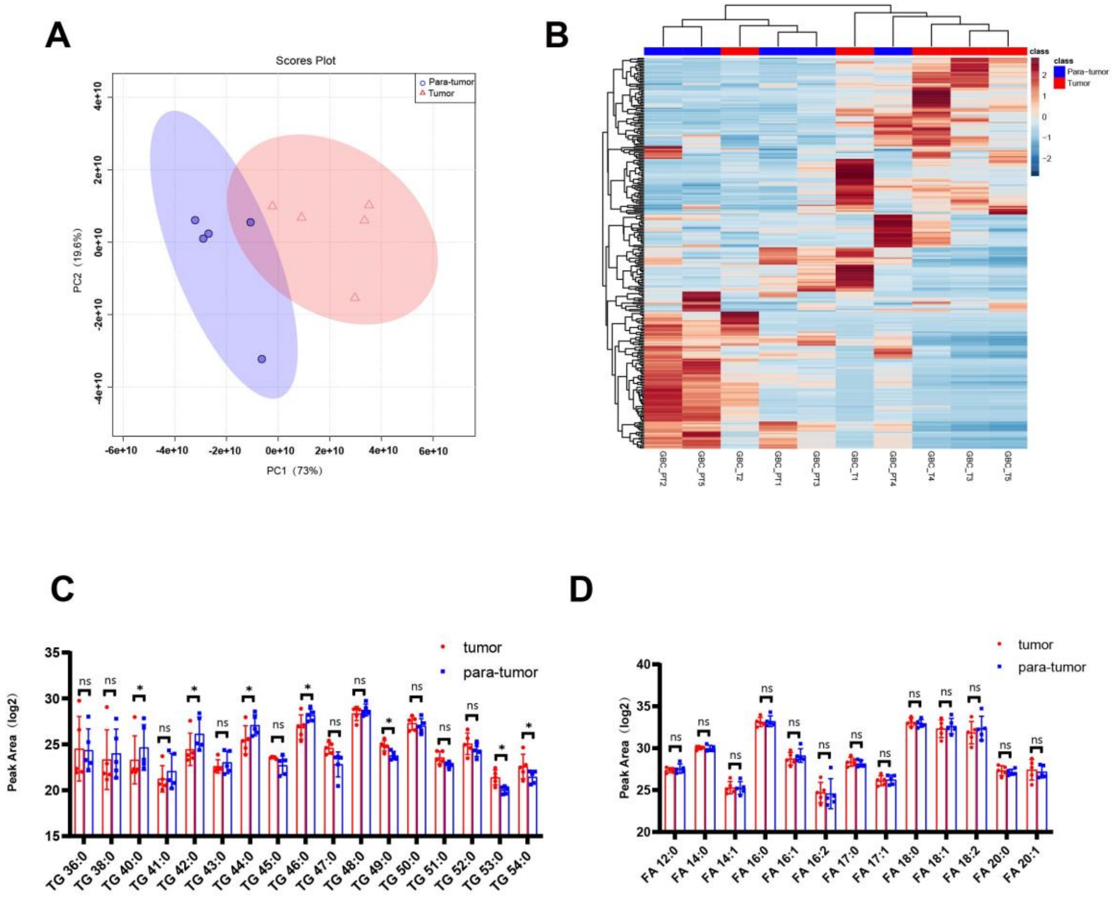


Fig S1

A Principal Component Analysis (PCA) of the lipomics in 5 paired GBC tissues.

B Hierarchical clustering analysis of the lipomics in 5 paied GBC tissues.

C,D Expression of different types of triglycerides and fatty acids in gallbladder carcinoma compared to adjacent tissues

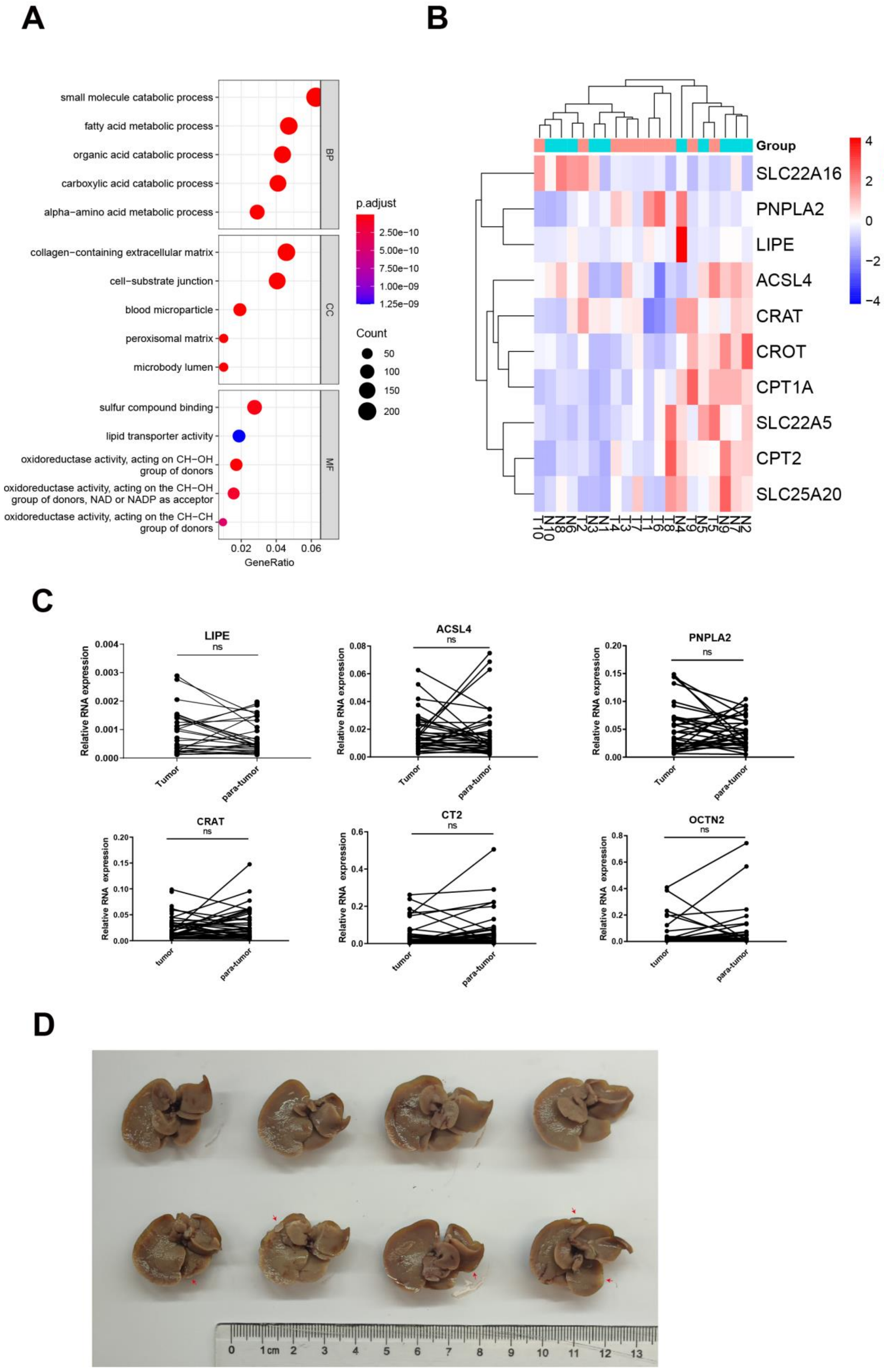
A
small molecule catabolic process
fatty acid metabolic process
organic acid catabolic process
carboxylic acid catabolic process
alpha−amino acid metabolic process
collagen−containing extracellular matrix
cell−substrate junction
blood microparticle
peroxisomal matrix
microbody lumen
sulfur compound binding
lipid transporter activity
oxidoreductase activity, acting on CH−OH group of donors
oxidoreductase activity, acting on the CH−OH group of donors, NAD or NADP as acceptor
oxidoreductase activity, acting on the CH−CH group of donors
BP
CC
MF
0.02 0.04 0.06
GeneRatio
p.adjust
2.50e−10
5.00e−10
7.50e−10
1.00e−09
1.25e−09
Count
50
100
150
200
B
Group
Normal
Tumor
SLC22A16
PNPLA2
LIPE
ACSL4
CRAT
CROT
CPT1A
SLC22A5
CPT2
SLC25A20
4
2
0
−2
−4
C
LIPE
ACSL4
PNPLA2
CRAT
CT2
OCTN2
ns
Relative RNA expression
Tumor
para-tumor
tumor
D
1cm

Fig S2

A GO enrichment analysis of differential genes in GBC tissues.

B FAO related genes in GSE139683.

C RNA expression levels of FAO metabolism related genes LIPE, ACSL4, PNPLA2, CRAT, CT2, OCTN2.

D The image of liver metastasis in mice with NOZ cells injected into spleen after 4 weeks of high-fat diet.

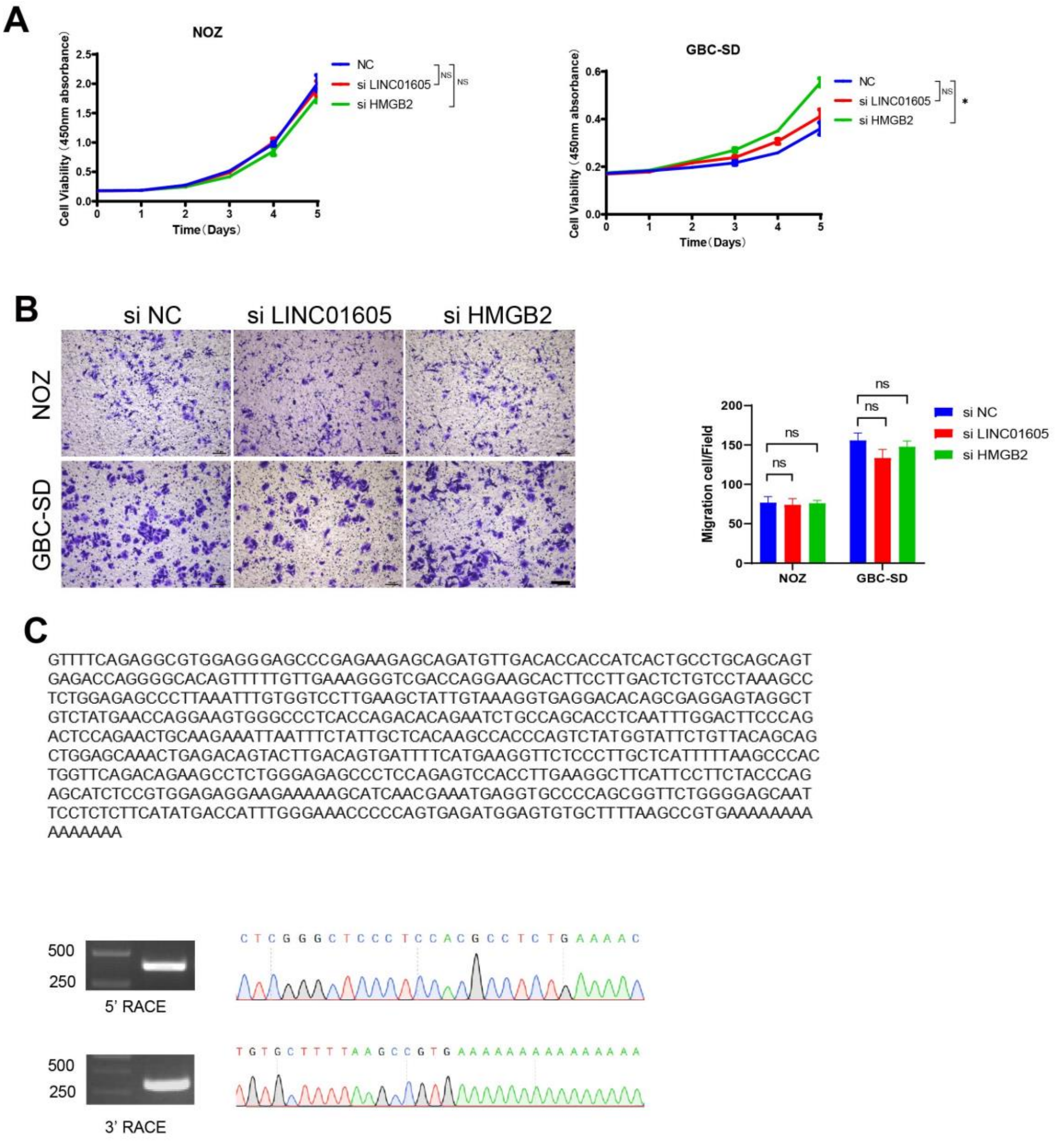


Fig S3

A Cell proliferation was detected after knockdown of LINC01605 and HMGB2 by CCK-8 assay.

B Detection of cell migration after knockdown of LINC01605 and HMGB2 by transwell assay.

C Sequence identification of full length lncBCL2L11 in NOZ cells through 5′ and 3′ rapid amplification of cDNA ends (RACE) assays.

**A**

| Label | Strand | Frame | Start | Stop | Length (nt \| aa) |
|---|---|---|---|---|---|
| ORF2 | + | 1 | 211 | 360 | 150 \| 49 |
| ORF3 | + | 3 | 327 | 464 | 138 \| 45 |
| ORF4 | - | 2 | 516 | 382 | 135 \| 44 |
| ORF1 | + | 1 | 37 | 153 | 117 \| 38 |
| ORF5 | - | 3 | 491 | 408 | 84 \| 27 |

**B**

Result for species name : hg19 with job ID :1651744932

| Data ID | Sequence Name | RNA Size | ORF Size | Ficket Score | Hexamer Score | Coding Probability | Coding Label |
|---|---|---|---|---|---|---|---|
| 0 | AC068491.2 | 631 | 150 | 0.7184 | -0.0737165304026 | 0.0086373274912515 | no |

**C**

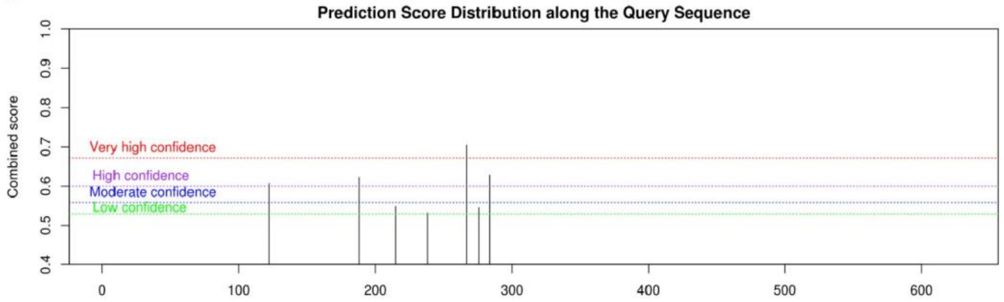


| # | Position | Sequence context | Score(binary) | Score(knn) | Score(spectrum) | Score(combined) | Decision |
|---|---|---|---|---|---|---|---|
| 1 | 122 | UCGAC CAGGA AGCAC<br>UUCCU UGACU CUGUC<br>CUAAA GCCUC UGGAG | 0.532 | 0.437 | 0.730 | 0.606 | m6A site (**High** confidence) |
| 2 | 188 | UGAAG CUAUU GUAAA<br>GGUGA GGACA CAGCG<br>AGGAG UAGGC UGUCU | 0.560 | 0.335 | 0.743 | 0.622 | m6A site (**High** confidence) |
| 3 | 215 | GCGAG GAGUA GGCUG<br>UCUAU GAACC AGGAA<br>GUGGG CCCUC ACCAG | 0.474 | 0.468 | 0.659 | 0.548 | m6A site (**Low** confidence) |
| 4 | 238 | CCAGG AAGUG GGCCC<br>UCACC AGACA CAGAA<br>UCUGC CAGCA CCUCA | 0.418 | 0.531 | 0.690 | 0.532 | m6A site (**Low** confidence) |
| 5 | 267 | AUCUG CCAGC ACCUC<br>AAUUU GGACU UCCCA<br>GACUC CAGAA CUGCA | 0.717 | 0.658 | 0.694 | 0.705 | m6A site (**Very high** confidence) |
| 6 | 276 | CACCU CAAUU UGGAC<br>UUCCC AGACU CCAGA<br>ACUGC AAGAA AUUAA | 0.472 | 0.416 | 0.663 | 0.545 | m6A site (**Low** confidence) |
| 7 | 284 | UUUGG ACUUC CCAGA<br>CUCCA GAACU GCAAG<br>AAAUU AAUUU CUAUU | 0.612 | 0.411 | 0.678 | 0.628 | m6A site (**High** confidence) |

Fig S4

A Prediction of potential encoding proteins of lncBCL2L11 by ORF finder.

B Coding potential of lncBCL2L11 predicted by CPAT.

C Prediction of m6A methylation sites in lncBCL2L11 using SRAMP.

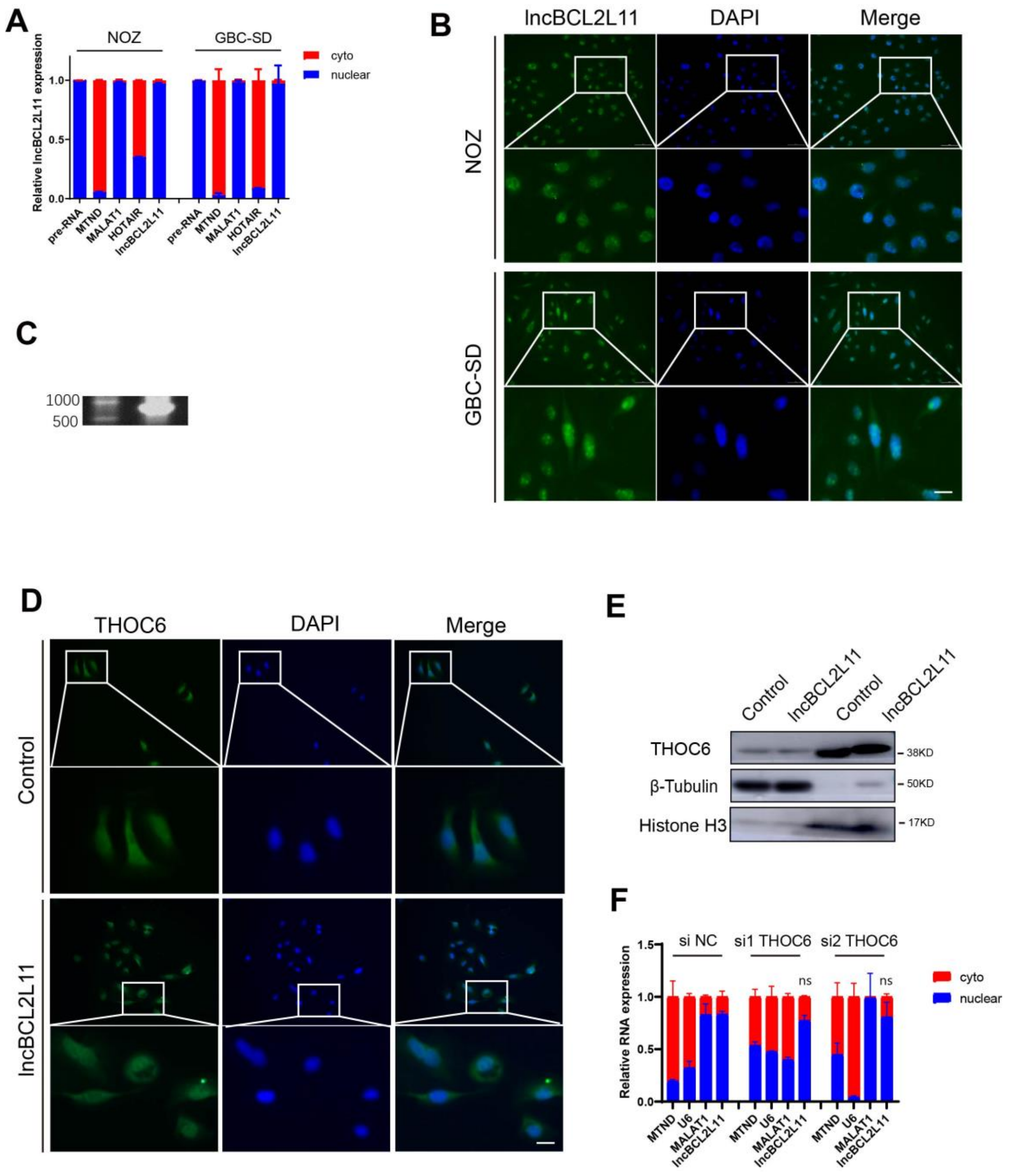


Fig S5

A Localization of lncBCl2L11 in NOZ cells and GCB-SD cells by RT-PCR after Isolation of cytoplasmic and nuclear RNA, MALAT1 served as a reference of cytoplasm and MTND, HOTAIR served as a reference of nuclear.

B Fluorescence in Situ Hybridization (FISH) assay (green) used to examine the expression and location of lncBCL2L11 in NOZ and GBC-SD cells.

C Identification of biotin-labeled lncBCL2L11 synthesized in vitro.

D Localization of THOC6 in lncBCL2L11-overexpressing GBC-SD cells by Immunofluorescence assay.

E Protein detection of THOC6 in isolated cytoplasmic and nuclear protein of lncBCL2L11-overexpressing GBC-SD cells. Tubulin was used as the internal reference of cytoplasm and Histone H3 as the internal reference of nucleus.

F Detection of lncBCL2L11 in nucleus and cytoplasm of THOC6 knockdown NOZ cells by RT-PCR.

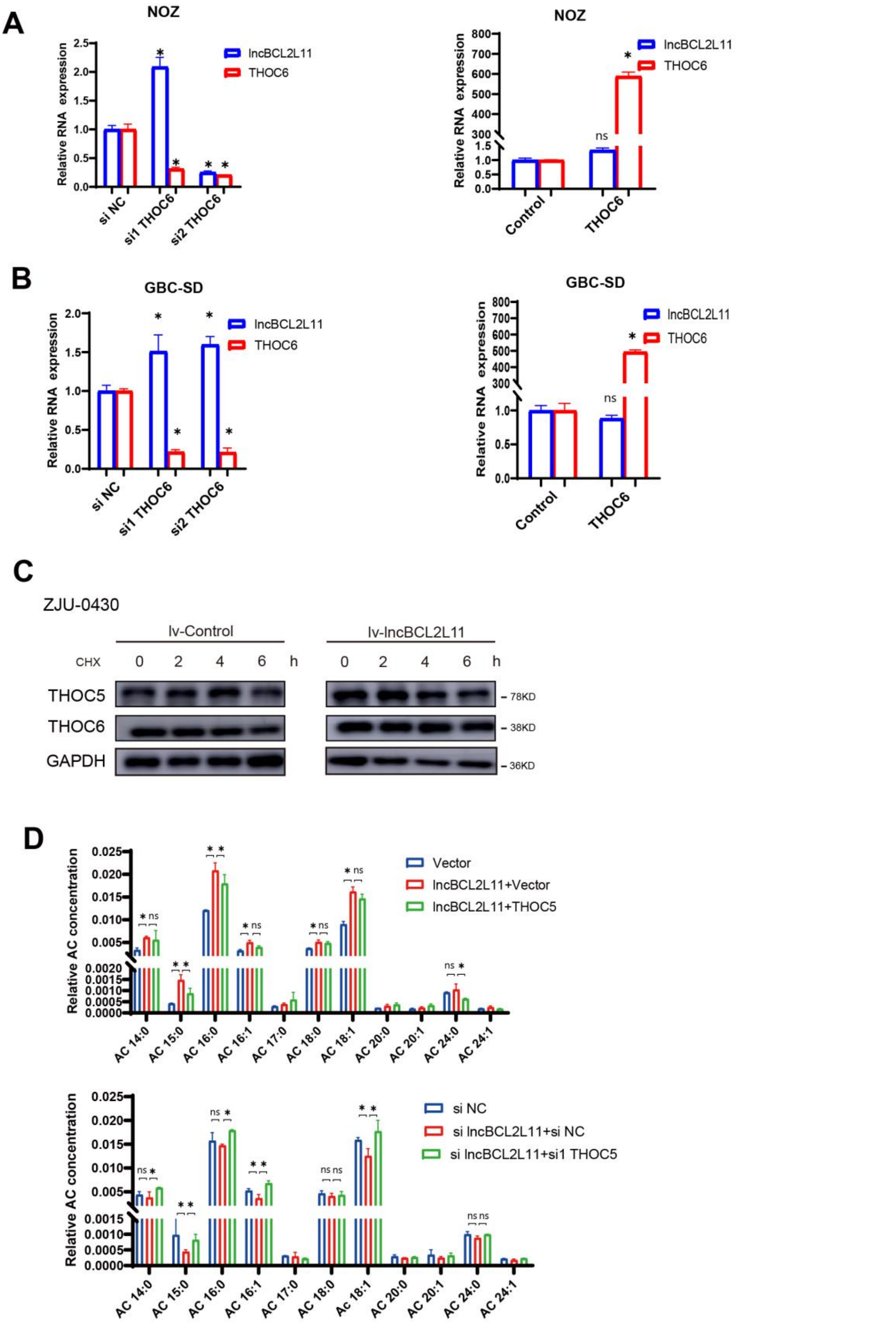


Fig S6

A,B Alteration of lncBCL2L11 expression after knockdown of THOC6 in NOZ and

GBC-SD cells.

C Degradation rate of THOC5 and THOC6 in lncBCL2L11-overexpressing ZJU-0430 cells.

D Detection of different acylcarnitine concentrations in indicated cells.

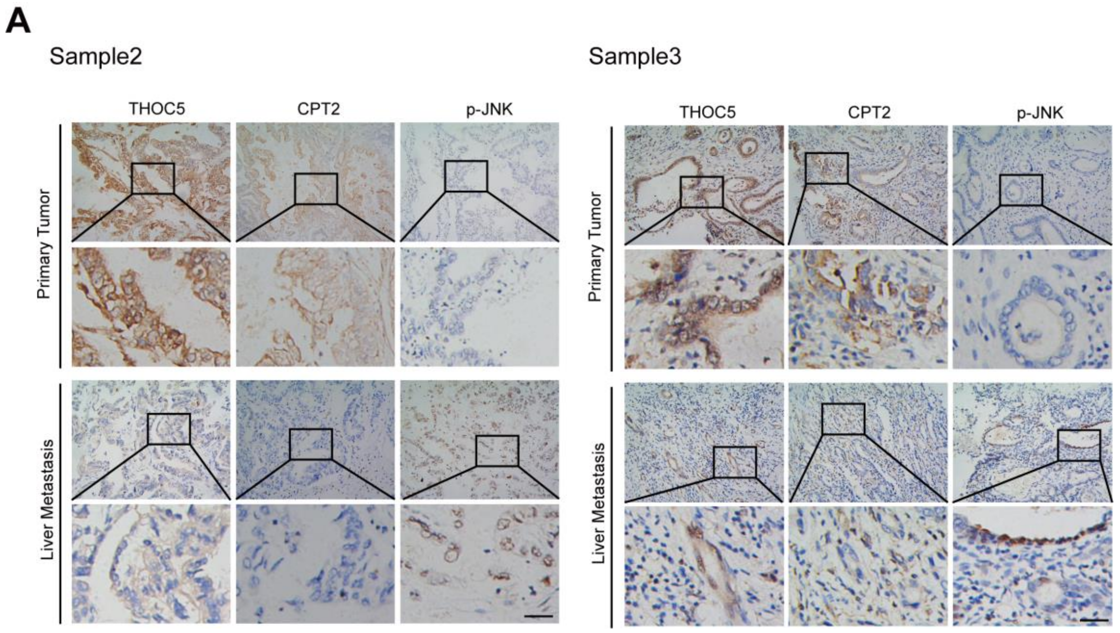


Fig S7

A Representative images of THOC5, CPT2, p-JNK in primary tumor and liver metastases. Scale bars, 100 μm.
